# Identification of Immune-Related lncRNA Signature to Predict Prognosis and Immunotherapeutic Efficiency in Bladder Cancer

**DOI:** 10.3389/fonc.2020.542140

**Published:** 2021-01-20

**Authors:** Lianghao Zhang, Longqing Li, Yonghao Zhan, Jiange Wang, Zhaowei Zhu, Xuepei Zhang

**Affiliations:** ^1^ Department of Urology, The First Affiliated Hospital of Zhengzhou University, Zhengzhou, China; ^2^ Department of Orthopedics, The First Affiliated Hospital of Zhengzhou University, Zhengzhou, China

**Keywords:** bladder cancer, The Cancer Genome Atlas, immune-related long noncoding RNA, prognosis, tumor immune microenvironment

## Abstract

**Purpose:**

Identify immune-related lncRNA (IRL) signature related to the prognosis and immunotherapeutic efficiency for bladder cancer (BLCA) patients.

**Methods:**

A total of 397 samples, which contained RNA-seq and clinical information from The Cancer Genome Atlas (TCGA) database, were used for the following study. Then the Lasso penalized Cox proportional hazards regression model was used to construct prognostic signature. According to the optimal cut-off value determined by time-dependent ROC curve, low and high-risk groups were set up. One immunotherapy microarray dataset as validation set was used to verify the ability of predicting immunotherapy efficacy. Furthermore, more evaluation between two risk groups related clinical factors were conducted. Finally, external validation of IRL-signature was conducted in Zhengzhou cohort.

**Result:**

Four IRLs (*HCP5, IPO5P1, LINC00942*, and *LINC01356*) with significant prognostic value (P<0.05) were distinguished. This signature can accurately predict the overall survival of BLCA patients and was verified in the immunotherapy validation set. IRL-signatures can be used as independent prognostic risk factor in various clinical subgroups. According to the results of GSVA and MCP algorithm, we found that IRL-signature risk score is strikingly negative correlated with tumor microenvironment (TME) CD8+T cells and Cytotoxic lymphocytes infiltration, indicating that the better prognosis and immunotherapy might be caused partly by these. Then, the results from the TIDE analysis revealed that IRL could efficiently predict the response of immunotherapy in BLCA. External validation had similar results with TCGA-BLCA cohort.

**Conclusions:**

The novel IRL-signature has a significant prognostic value for BLCA patients might facilitate predicting the efficacy of immunotherapy.

## Introduction

Bladder cancer has become the second most commonly seen malignancies of the urinary system in the United States of America (1). Despite the establishment of several novel treatment strategies, BLCA remains an important medical concern (2). An American research estimated that 80,470 new BLCA patients and 1,767 deaths in 2019 (3). Once the tumor has developed to a locally advanced or metastatic stage, surgical treatment combined with general chemotherapy is inadequate for the treatment of BLCA (4, 5). According to research, the response rate for novel immunotherapy and immune checkpoint inhibitors (ICIs) is 30% or less (6). Therefore, there is still an urgent clinical demand to discovery molecular biomarkers by molecular profiling for BLCA.

Long noncoding RNA (lncRNA) is a kind of noncoding RNA that has more than 200 nucleotides in length. As the flourish of the study in lncRNAs, lots of evidences show that immune-related lncRNAs (IRLs) play a significant role in tumor microenvironment (TME) (7). If we could discovery their protentional immune molecular signal and motivated them, the immune cells in TME could suppress tumor progression, recurrence, and metastasis (8, 9). Gene polymorphisms in lncRNA have been linked to increased risk in many kinds of cancer, such as prostate cancer (10), gastric cancer (11), breast cancer (12), ovarian cancer (13).

In this research, we tend to discover a novel IRLs signature by a LASSO penalized Cox regression (iterations = 1000) analysis. This algorithm has produced reliable results in various studies (14, 15). The four lncRNAs we identified to construct signature are all reported for the first time in bladder cancer. The IRL-signature composed of them has a strong predicted ability in overall survival (OS) compared with general clinical feature for BLCA patients. Furthermore, we selected two representative cohorts including a cohort of patients treated with ICIs to validate our signature model. Surprisingly, the IRL-signature have a great correlation in TME immune cell infiltration and the treatment response of ICIs. In summary, this novel IRL-signature compared with previous studies have a more guiding significance in predicting patient prognosis and the effectiveness of ICI immunotherapy.

## Materials and Methods

### Data Collection

Normalized RNA-Seq (FPKM format) data for BLCA, downloaded from The Cancer Genome Atlas (TCGA) database (https://cancergenome.nih.gov/), includes 414 tumor samples and 19 normal samples. Clinical data, include survival time, survival state, age, gender, were also derived from TCGA database and lacked complete data were excluded. Afterwards, samples with OS ≤30 days were excluded because of the factors of nonneoplastic death (16). A total of 397 samples were used as training set for the following study. Subsequently, with the help of R software’s “IMvigor” software package to obtain the IMvigor data set, we acquired data of BLCA tumor patients treated with *PD-L1* ICI immunotherapy (EGAS#00001002556), and set it as a validation set (n=348).

### Human Bladder Cancer Cell Lines Culture

Human bladder cancer cell lines (T24, 5637, RT-4, UM-UC-3, and HT1376) and human normal urothelium cell line (SV-HUC-1) were obtained from the Stem Cell Bank, Chinese Academy of Sciences in Shanghai, China. The SV-HUC-1 cell was cultured in Dulbecco’s Modified Eagle Medium (Invitrogen, Carlsbad, CA, USA) plus 10% fetal bovine serum. All bladder cancer cells were cultured in RPMI-1640 Medium (Invitrogen, Carlsbad, CA, USA) plus 10% fetal bovine serum. Corresponding plates were placed at 37°C with a humidified atmosphere of 5% CO2 in incubator (17).

### Quantitative Real-Time PCR Analysis

The total RNA of the cells and tissue samples were extracted using the TRIzol reagent (Invitrogen, Carlsbad, CA, USA). The detailed primer sequences included in this study are shown in **Table S1**. Quantitative real-time PCR (qRT-PCR) was performed using the ABI PRISM 7000 Fluorescent Quantitative PCR System (Applied Biosystems, Foster City, CA, USA) according to the manufacturer’s instructions and normalized to GAPDH small nuclear RNA. Experiments were repeated at least 3 times (18).

### Identification of Immune-Related lncRNA

First, we obtained immune‐related genes from the ImmPort database (https://immport.niaid.nih.gov). Then the lncRNA profile was extracted from mRNA expression data by R software. Pearson correlation analysis was conducted to identify IRLs between immune‐related genes and lncRNAs. At last, 70 IRLs were selected by the criteria, |R| > 0.8 and P-value < 0.001, in TCGA-BLCA dataset.

### Identification of Prognostic Signature-Based Immune-Related lncRNAs

To identify IRLs for using in clinical settings, a lasso penalized Cox regression (iterations = 1000) was applied using the “glmnet” R package to establish a more stable prognostic model (17). We screened for IRLs using 500 repetitions and used the derived coefficients to calculate the risk score:

Risk score=coef gene (1)×exprgene (1)+coef gene (2)× exprgene (2)+⋯+coef gene (n)× exprgene (n)

A time-dependent receiver operating characteristic (ROC) curve analysis was used to determine the optimal cut-off value for IRL-s training group by using the “survivalROC” R package. Based on the cut-off value of IRL-s, patients were divided into high-risk and low-risk groups. The log-rank test was used to evaluate the OS difference between the low-risk and the high-risk patients, and the Kaplan–Meier (KM) survival curve was derived by using R package “survminer”.

### Validation of Prognostic Immune-Related lncRNA-Signature by PD-L1 Blockade Treated Cohort

After constructing prognostic IRL-signature by TCGA-BLCA training set, IMvigor data set was used as a validation set to verify predictions of the effectiveness of ICI immunotherapy.

### Analysis of Clinical Features of Immune-Related lncRNA-Signature-Based Low- and High-Risk Patients

We compared predictive value of IRL-signature with other Clinical features (age, WHO Stage, AJCC-T stage, AJCC-N stage and grade) by univariate, multivariate Cox regression analysis and multi-index ROC curve. The predictive value of IRL-signature in different clinical subgroups has also been explored.

### Bioinformatics Analysis

Gene Set Variation Analysis (GSVA) is an unsupervised gene set enrichment method that can estimate the scores for certain pathways or markers with in a sample population (19). We downloaded the “ Hallmark “ gene sets from the Molecular Signatures Database (http://software.broadinstitute.org/gsea/index.jsp) (16) for GSVA through package “GSVA” in R. The “MCPcounter” package in R was used for analysis of microenvironment cell populations (MCPs) and quantification of immune cells from transcriptomic data. The result was shown by the package “pheatmap” in R. Subsequently, package “limma” in R was used to identify the pathways with the most significant differences between patients in the signature group.

### Estimation of Immune-Checkpoint Inhibitors Response

Different expression of seven immune checkpoints were detected between low and high-risk patients by box plot. Furthermore, the immune checkpoint inhibitor (ICI) response was assessed by using the tumor immune dysfunction and exclusion (TIDE) algorithm (http://tide.dfci.harvard.edu/) (18).

### Construction of a Nomogram

The clinical characteristics of the TCGA-BLCA cohort were combined with the IRL- signature to construct a nomogram by using the “rms” R package. We used the C index to evaluate the discriminative power and draw a calibration chart to evaluate the accuracy of the nomogram.

### External Validation in Zhengzhou Cohort

A total of 54 BLCA patients treated in The First Affiliated Hospital of Zhengzhou University were served as external validation cohort. The tumor tissues were obtained at the first-time surgery. The study has been approved by the Ethics Committee of The First Affiliated Hospital of Zhengzhou University. The extrication of their total RNA and Quantitative real-time PCR analysis were performed following standard protocols as previously mentioned. The relative lncRNA expression levels were calculated by 2^−Δct^ method. Risk score was calculated with the previous following formula of signature. Ultimately, univariate and multivariate Cox regression analysis, time-dependent ROC curve and KM survival analysis were performed for the validation in Zhengzhou cohort (n = 54).

### Statistical Analysis

We utilize R software (v4.0.0: http://www.r-project.org) to conduct statistical analysis. Chi-square test and t test were used to evaluate qualitative variables and quantitative variables, respectively. Time-dependent ROC curve and c-index was utilized to assess the prognostic value based on the lncRNA signature. Delong’s Z-test was utilized to compare the AUC and c-index between the signature and nomogram (20). The P-value < 0.05 indicated statistical significance.

## Result

### Construction and Validation of Prognostic Immune-Related lncRNA-Signature

**Figure 1:**
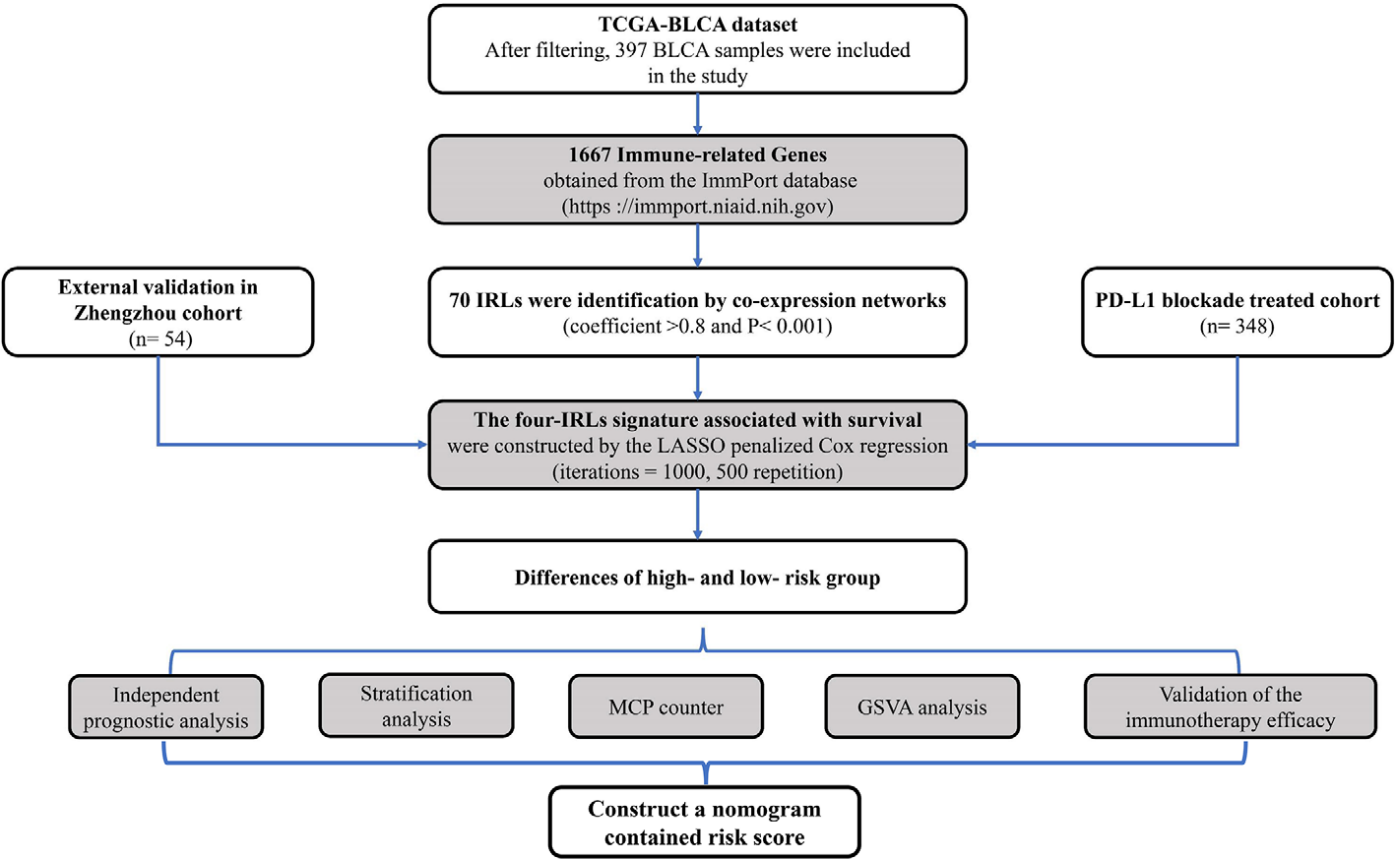
The flowchart describes the construction and validation of IRL-signature.

In order to make our study procedure clearer, the workflow is shown in **Figure 1**. After the above-mentioned criteria filtering, a total of 397 TCGA-BLCA samples were included in our study. Subsequently, 70 IRLs were identified by co-expression networks (coefficient >0.8 and P< 0.001). Lasso-penalized multivariate Cox proportional hazards modeling was conducted on abovementioned 70 IRLs. After 1,000 iterations, four IRL-signature accommodated optimal survival prediction in the training set more than 500 times each. The four lncRNAs was used for following study (**Table 1**). Based on these four lncRNAs (HCP5, IPO5P1, LINC00942, and LINC01356) and their derived coefficients, we established a risk score with the following formula:

Risk score=(−0.026×Expression HCP5)+(−0.104×Expression IPO5PI)+(0.015×Expression LINC00942)+(0.024×Expression LIN01356).

**Table 1:** Description of the four modeling genes.

Gene symbol	Entrez ID	Coefficient	Description
*HCP5*	10866	*-0.026*	HLA complex P5
*IPO5P1*	100132815	*-0.104*	NA
*LINC00942*	100292680	*0.015*	Long Intergenic Non-Protein Coding RNA 942
*LINC01356*	100996702	*0.024*	Long Intergenic Non-Protein Coding RNA 1356

**Figure 2:**
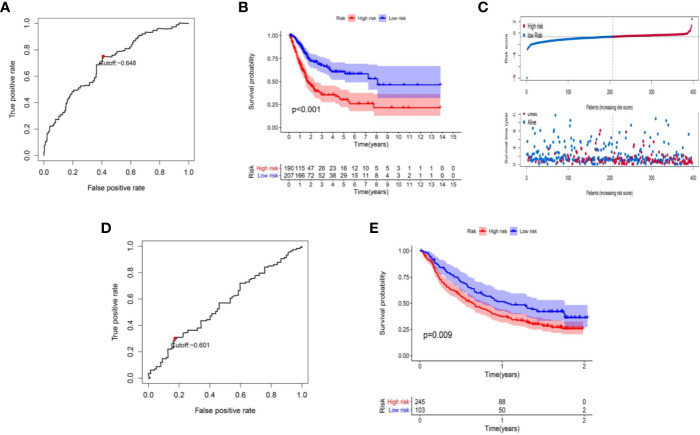
Construction of IRL-based signature. **(A)** Time-dependent ROC curve of IRL-signature in TCGA-BLCA cohort. The optimal cut-off value of LncRNA signature is -0.648, and patients are divided into high-risk group and low-risk group according to the cut-off value. **(B)** Kaplan–Meier curves of overall survival according to IRL-signature groups in the TCGA cohort. **(C)** Distribution of risk score, survival status of patients in in TCGA-BLCA cohort. **(D)** Time-dependent ROC curve of IRL-signature in IMvigor dataset. The optimal cut-off value of IRL-signature is -0.648, and patients are divided into high-risk group and low-risk group according to the cut-off value. **(E)** Kaplan–Meier curves of overall survival according to IRL-signature groups in the IMvigor dataset.

**Figure 3:**
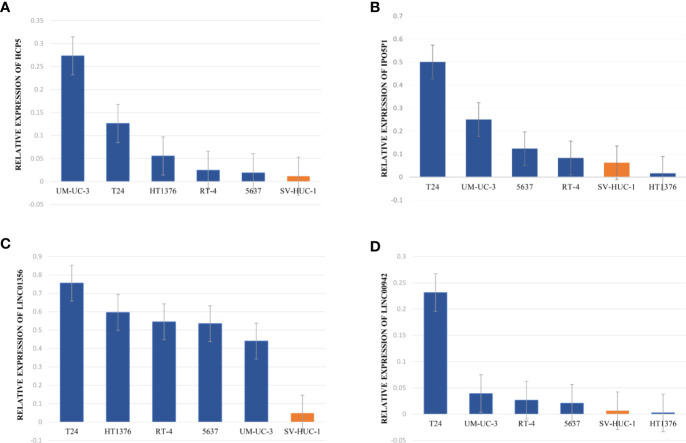
Upregulated expression of four selected lncRNAs in normal urothelium cell compared with BLCA and cell lines. **(A–D)** qRT-PCR analysis indicates the relative expression level of *HCP5* **(A)**, *IPO5P1* **(B)**, LINC01356 **(C)**, LINC00942 **(D)** in distinct BLCA cell lines (T24, 5637, RT-4, UM-UC-3, and HT1376) and normal immortalized urothelium cell SV-HUC-1. The GAPDH allele is used as a loading control.

The data of risk score of TCGA-BLCA samples were summarized in **Table S2**. The optimal cut-off of the IRL-signature between high- and low-risk group was set at -0.648 using time-dependent ROC analysis (**Figure 2A**). As shown in **Figure 2B**, low-risk patients have a better prognosis than high-risk (P<0.001). Furthermore, in the *PD-L1* immunotherapy treated validation cohort (EGAS#00001002556), we used the time-dependent ROC analysis to determine the optimal cut-off whose value was -0.601 (**Figure 2D**). In the PD-L1 immunotherapy treated validation cohort, the low-risk group also has a better prognosis than the high-risk group (P=0.009, **Figure 2E**). In addition, we measured gene expression in human bladder cancer cell and normal urothelium cell line, as shown in the **Figures 3A–D**, the expression levels of these four IRLs are upregulated in bladder cancer cell lines (T24, 5637, RT-4, UM-UC-3, and HT1376), compared with normal urothelium cell line (SV-HUC-1).

### Independent Prognostic Analysis of Immune-Related lncRNA-Signature in Bladder Cancer

We explored whether IRL-signature can be an independent prognostic factor in BLCA by univariate Cox regression and multivariate Cox regression analysis. The result of univariate Cox regression shown risk score can be responsible for OS just like other clinical indicators (Age, WHO-Stage, AJCC-T stage and AJCC-N stage; **Figure 5A**). Through multivariate Cox regression analysis, we found that IRL-signature risk score has greater correlation with OS than others (**Figure 5A**). In addition, according to time-dependent ROC analysis, IRL-signature showed an accuracy in predicting OS in TCGA-BLCA cohort and the area under curve (AUC) of ROC was 0.707 at 3 years (**Figure 5B**).

### Correlation Between Immune-Related lncRNA-Signature and Clinical Features

**Figure 4:**
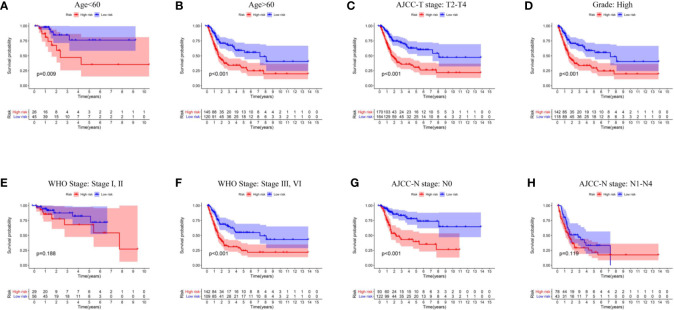
Kaplan–Meier curves analyses of clinical subgroups in TCGA cohort **(A)** Age < 60 years, **(B)** Age > 65 years, **(C)** AJCC-T stage: T2-T4, **(D)** Grade: High, **(E)** WHO Stage: Stage I, II, **(F)** WHO Stage: Stage III, VI, **(G)** AJCC-N stage: N0, **(H)** AJCC-N stage: N1–N4. WHO, World Health Organization; AJCC, American Joint Committee on Cancer.

**Figure 5:**
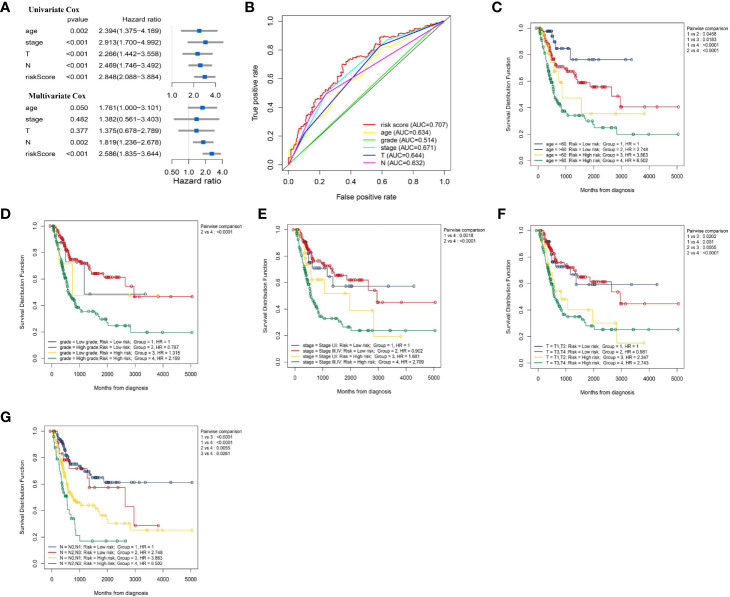
Evaluate whether lncRNA signature is an independent prognostic factor and relationship between risk score and clinical characteristics. **(A)** Forest plot of univariate and multivariate Cox regression results of lncRNA signature and clinical characteristics. **(B)** ROC curve of clinical characteristics and signature risk score for 3-year OS. Two factors Kaplan–Meier curves of overall survival for patients in TCGA cohort stratified by IRL- signature, age **(C)**, grade **(D)**, WHO stage **(E)**, AJCC-T stage **(F)**, and AJCC-N stage **(G)**. ROC, receiver operating characteristic; WHO, World Health Organization; AJCC, American Joint Committee on Cancer; AUC, area under the curve.

The summary of clinical characteristics of TCGA-BLCA was shown in **Table 2**. We further analyzed the relationship between IRL-signatures and clinical characteristics. We divided patients into different subgroups based on clinical variables. Because the TCGA database include only two patients with non-muscular invasion and fewer low-grade patients, we excluded these. As shown in **Figures 4A–H**, IRL-signatures can predict the OS of patients in different subgroups (Age<60, Age>60, AJCC-T2-T4, High Grade, WHO Stage III, VI, and AJCC-N0). In addition, based on clinical variables and IRL-signature, we divided the patients in the TCGA data set into four groups for two-factor KM analysis. As shown in **Figures 5C–G**, our results showed that there was no significant difference in overall survival between the low-grade and high-grade groups of patients in the IRL signature low-risk group. In the high-grade group, the overall survival rate of the low-risk group is higher than that of the high-risk group. This situation also occurs in the two WHO-Stage subgroups. This also explains why patients with similar clinical characteristics show completely different clinical outcomes despite undergoing the same treatment.

**Table 2:** Summary of clinical characteristics of TCGA-BLCA patient data sets in the study.

Characteristic	TCGA-BLCA data set (n = 397)
**Vital status, n (%)**	
Alive	244 (61.5)
Dead	153 (38.5)
Age, n (%)	
<60	86 (21.7)
≥60	311 (78.3)
**Grade, n (%)**	
Low Grade	19 (4.8)
High Grade	375 (94.5)
Unknow	3 (0.7)
**WHO-Stage, n (%)**	
I	2 (0.5)
II	125 (31.5)
III	138 (34.8)
VI	130 (32.7)
Unknow	2 (0.5)
**AJCC-T stage, n (%)**	
T0	1 (0.3)
T1	3 (0.8)
T2	114 (28.7)
T3	190 (47.9)
T4	57 (14.6)
Unknow	32 (8)
**AJCC-N stage, n (%)**	
N0	230 (57.9)
N1	44 (11.1)
N2	75 (18.9)
N3	7 (2)
NX	36 (9)
Unknow	5 (1)

### Functional Evaluation of Immune-Related lncRNA-Signature

**Figure 6:**
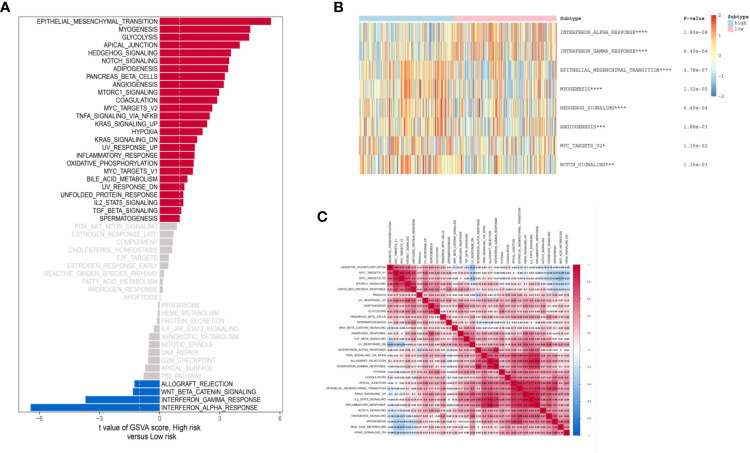
Biological function of two groups of patients. **(A)** Bar plot of “Hallmark” pathway score calculated by GSVA for two groups of patients. Heat map of GSVA analysis between two risk groups. **(B)** Heat map of most valuable pathway. **(C)** Correlation heatmap between each pathway. *P < 0.05, ***P < 0.005, ****P < 0.001.

Based on the “Hallmark” gene sets, we utilized the GSVA to explore the IRL-signature related functional annotation. As shown in bar plot and heatmap, we found that the IRL high-risk group were enriched in epithelial-mesenchymal transition (EMT), myogenesis, glycolysis, and angiogenesis pathways. The immune-related interferon alpha response and interferon gamma response pathways are enriched in the low-risk group (**Figures 6A, B**). Subsequently, we draw a correlation heatmap between various pathways (**Figure 6C**).

### Correlation of the Two Groups With Immune Infiltration

**Figure 7:**
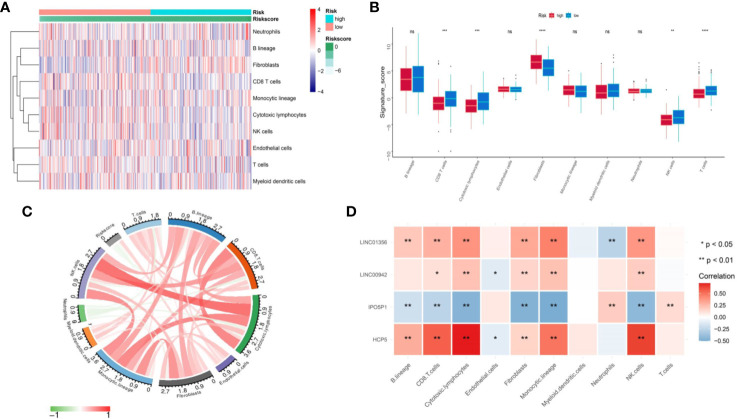
Assess the difference in immune infiltration between the two groups. **(A)** Differences of 10 cell abundances calculated by MCP-counter method between two groups of patients. **(B)** Box plot of ten gene sets calculated by MCP-counter. **(C)** The correlation between risk score and ten TME infiltration cells. **(D)** Correlation matrix of IRL and ten TME infiltration cells. The blue indicated positive correlation and yellow indicated negative correlation. ns P ≥ 0.05, *P < 0.05, **P < 0.01, ***P < 0.001, ****P < 0.0001.

MCP counter method was used to estimate the TME immune cells infiltration in BLCA. As indicated in figure, CD8 T cells, cytotoxic lymphocytes, T cells and NK (natural killer) cells have higher infiltration in TME of low-risk than high-risk (**Figures 7A, B**). The **Figure 7C** illustrated that risk score have a negative relation with Fibroblasts and positive relation with CD8+T cells, Cytotoxic lymphocytes, NK cells and Neutrophils (**Figure 7C**). Correlation analysis between four genes (HCP5, IPO5P1, LINC00942 and LINC01356) constituting the signature and immune cells shows that HCP5, IPO5P1, LINC00942 and LINC01356 are positively correlation, and IPO5P1 is negatively correlation (**Figure 7D**).

### Distinct Sensitivity of Immune Checkpoint Inhibitors for Two Risk Groups of Bladder Cancer

**Figure 8:**
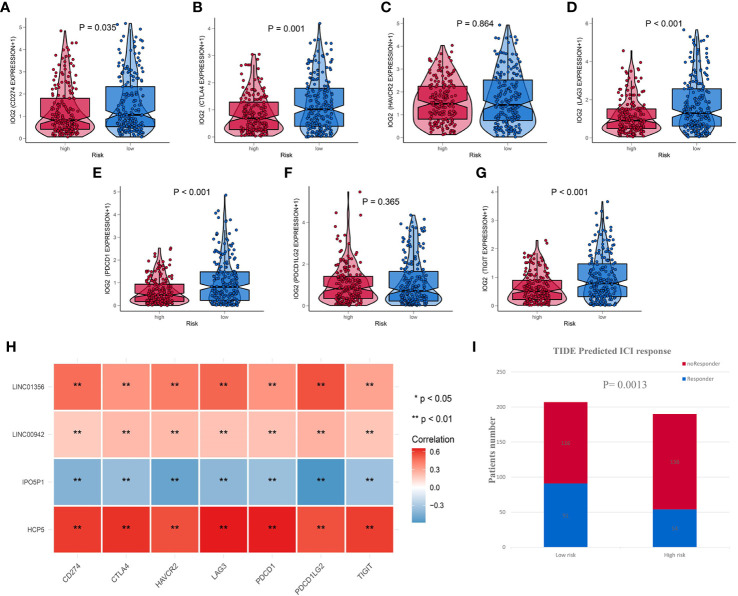
IRL-signature was efficient in prediction the immunotherapeutic benefit in BLCA **(A–G)** Box-Violin plots visualized the correlation between Risk score and immune-checkpoint-relevant genes, CD274 **(A)** CTLA-4 **(B)**, HAVCR2 **(C)**, LAG3 **(D)**, PDCD1 **(E)**, PDCD11LG2 **(F)**, and TIGIT **(G)**. **(H)** Correlation heatmap of four immune-related lncRNAs and seven immune-checkpoint-relevant genes. **(I)** The distribution of immunotherapeutic response in indicated groups stratified by IRL-signature in TCGA-BLCA cohort based on the TIDE algorithm. **P < 0.01.

Due to IRL-signature can identify patients with better prognosis in the immunotherapy verification set, we next wonder to further verify the stability of the result. Firstly, we selected seven immune-checkpoints, including *CD274 (PD-L1), CTLA-4, HAVCR2 (TIM-3), LAG-3, PDCD1 (PD-1), PDCD1LG2*, and *TIGHT*, to analyze the difference between two risk groups. We found that six immune checkpoints were upregulated in IRL low-risk group (**Figures 8A–G**). When it comes to single IRL, the expression of *HCP5, LINC00942*, and *LINC01356* is positively correlated with the expression of immune checkpoints. While the expression of *IPO5P1* has a negative correlation (**Figure 8H**).

The TIDE algorithm, which was established to predict the ICI responders through transcriptomic data, was used to explore whether IRL-signature could predict immunotherapeutic benefit in TCGA-BLCA cohort. The results of every patient were shown in **Table S3**. The output revealed that the number of ICI responders were significantly higher in IRL- low-risk patients (n=91) compared with IRL- high-risk patients (n=54) (Chi-square test, P = 0.0013; **Figure 8I**). The IRL risk-score was robustly negative correlated with the immunotherapy response in BLCA patients. In IMvigor validation set, the signature can identify patients who have benefited from immunotherapy. All the results indicate that the signature may be used as a predictor of immunotherapy efficacy.

### Construction Nomogram Based on Immune-Related lncRNA-Signature

**Figure 9:**
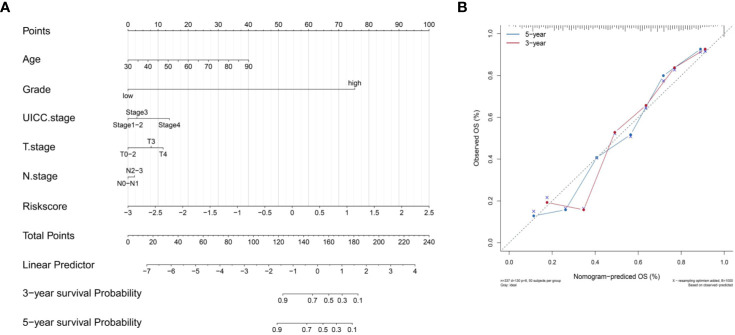
Construct nomogram. **(A)** Nomograms for predicting the probability of patient mortality based on IRL-signature and clinical variables. **(B)** The calibration plot for internal validation of the nomogram.

We constructed a nomogram based on the clinical variables and IRL-signatures of the TCGA dataset (**Figure 9A**). The results of the calibration chart show that the nomogram performance is the best in predicting the 5 years OS (**Figure 9B**). In the TCGA cohort, the nomogram C index was 0.737.

### External Validation of Immune-Related lncRNA-Signature in Zhengzhou Cohort

In order to make IRL-signature prognostic prediction ability more credible, we use the Zhengzhou cohort as an external validation set. Clinical features of enrolled BLCA patients are presented in **Table 3**. We performed qRT-PCR to measure the expression levels of the four lncRNAs and the risk scores of every patients were calculated with previous following formula of signature (**Table S4**).

**Table 3:** Summary of clinical characteristics of Zhengzhou external validation data set.

Characteristic	Zhengzhou data set (n = 54)
**Vital status, n (%)**	
Alive	30 (55.6)
Dead	24 (44.4)
Age, n (%)	
<60	21 (38.9)
≥60	33 (61.1)
**Grade, n (%)**	
Low Grade	33 (61.1)
High Grade	21 (38.9)
**Gender, n (%)**	
Female	17 (31.5)
Male	37 (68.5)
**AJCC-T stage, n (%)**	
T1	4 (7)
T2	16 (30)
T3	25 (46.3)
T4	9 (16.7)
**AJCC-N stage, n (%)**	
N0	19 (35.2)
N1	15 (27.8)
N2	14 (25.9)
N3	6 (11.1)

**Figure 10:**
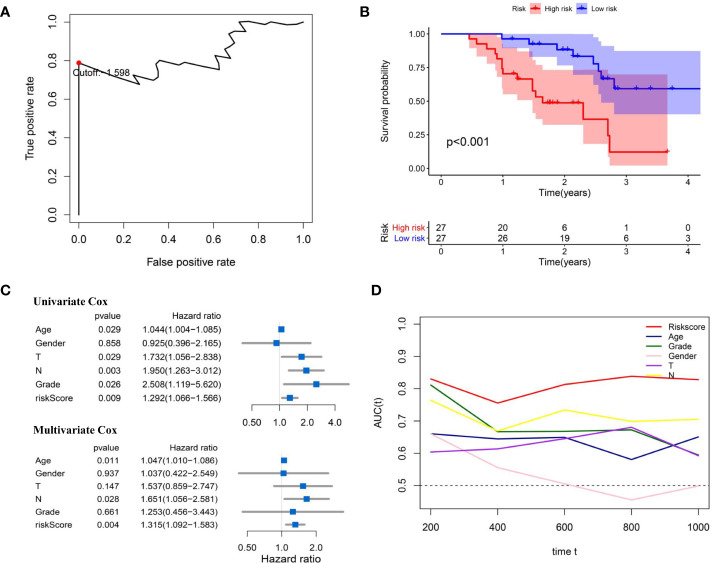
External validation of IRL-signature in Zhengzhou cohort. **(A)** Time-dependent ROC curve of IRL-signature in Zhengzhou cohort. The optimal cut-off value of LncRNA signature is -1.598, and patients are divided into high-risk group and low-risk group according to the cut-off value. **(B)** Kaplan-Meier survival curve of IRL-signature in Zhengzhou validation cohort. **(C)** Forest plot for univariate and multivariate Cox regression analysis. **(D)** Time-dependent ROC curves and AUC of IRL-signature based on Zhengzhou validation set.

As shown in **Figure 10A**, according to the optimal cut-ff value determined by time-dependent ROC curve (cut-off = -1.598) significant difference in OS between high and low risk groups (P<0.001, **Figure 10B**). Subsequently, univariate and multivariate Cox regression analysis revealed that risk score of the signature cloud be an independent prognostic factor (univariate Cox: HR = 1.292, 95%CI =1.066-1.566, P = 0.009; multivariate Cox: HR = 1.315, 95%CI =1.092-1.583, P = 0.004; **Figure 10C**). The time-dependent ROC curve indicated that the AUC of the risk score was higher at 1, 3 and 5 years, compared with other clinical features (**Figure 10D**).

## Discussion

With the continuous development of high-throughput sequencing technology, exploring specific mRNA expression, such as tumor microenvironmental genes and metabolic genes, can help us better identify tumor diversity and formulate personalized treatment strategies. The heterogeneity of tumor immunity contains multi-dimensional information about patient prognosis and treatment response. Therefore, based on the immune-related genes in the ImmPort database, our study identified 70 immune-related lncRNAs in bladder cancer patients through co-expression analysis. Subsequently, we used the TCGA cohort as a training set and identified four robust lncRNAs using LASSO penalized Cox regression analysis. Subsequently, these robust lncRNAs were used to construct signatures. Signatures identify patients with a high risk of death, and we have reached similar conclusions in the IMvigor data set. The four lncRNAs are all risk factors for bladder cancer. Further qRT-PCR results showed that compared with normal urothelial cells, except for the down-regulation of IPO5T1 and LNC00942 genes in the HT1376 cell line, the expression of other genes was upregulated in all cell lines. Subsequently, through univariate and multivariate Cox regression analysis, we found that IRL characteristics combined with other risk factors may still be independent prognostic factors. In addition, the signature is positively correlated with many malignant clinical features. Finally, we verified the prognostic value of signatures in a unique data set-Zhengzhou cohort.

Today, clinicopathological factors are still the most important guidelines in the diagnosis and treatment of bladder cancer. However, the prognosis of some patients with similar clinical characteristics is quite different. Therefore, we combined the signature with WHO stage, AJCC-T, N stage, and other clinicopathological factors to divide the patients into four groups. Our results show that the combination of signatures and clinicopathological factors can more accurately identify patients. For example, according to risk grouping and T grade, patients are divided into four different groups. High-risk patients in the T0-T1 classification have a worse prognosis, while patients in the low-risk group in the T3-T4 classification have a better prognosis. At the same time, combining risk groups with clinical pathological factors such as UICC stage, Grade, and N can more accurately identify high-risk patients.

Subsequently, we further explored the differences in the biological behavior of patients between the signature groups. We used GSVA to evaluate the biological behavior of TCGA-BLCA patients. The results of GSVA showed that there were significant differences in the biological behavior of patients between the signature groups. Subsequently, “limma” was used to identify the pathways with the most significant differences between patients in the signature group. Our results show that compared with the high-risk group, patients in the low-risk group have higher scores for the interferon gamma response and interferon alpha response pathways. On the contrary, in the high-risk group, Epithelial Mesenchymal Transition, Myogenesis, Hedgehog Signaling and Angiogenesis, which are thought to have immunosuppressive effects and play an important role in tumorigenesis, significantly increased their scores. Subsequently, we further evaluated the differences in immune cell infiltration between the signature group. Our results showed that the infiltration abundance of CD8 T cells, cytotoxic lymphocytes, NK cells and T cells was significantly higher in the low-risk group. The high-risk group had higher fibrocyte infiltration abundance. This is consistent with the results of GSVA to a certain extent.

Nowadays, immune checkpoint inhibitor therapy has been shown to produce durable clinical responses to patients with various advanced cancers, such as melanoma, non-small cell lung cancer, renal cell carcinoma and Hodgkin’s lymphoma (21–24). Currently, there are three *PD-L1* inhibitors and two *PD-1* inhibitors approved by the Food and Drug Administration for the treatment of bladder cancer (Atezolizumab, Avelumab, Durvalumab, Nivolumab, Pembrolizumab, and Ipilimumab). Unfortunately, only some patients can benefit from this treatment strategy (25, 26). This new treatment method raises questions about how to identify patients who respond to the therapy. Previous studies have shown that the use of immunohistochemistry to detect the expression of *PD-L1* on the surface of tumor cells can be used as a predictor of patient response to treatment (27). Unfortunately, the predictive power of this method is limited, because not all *PD-L1* positive patients respond well (28, 29). In addition, recent studies have shown that tumor mutation burden (TMB) is also expected to be a marker for identifying patients who can benefit from immunotherapy. As a marker for predicting immunotherapy response, TMB has shown encouraging results in non-small cell lung cancer and melanoma (30, 31). However, in bladder cancer, the relationship between TMB and immunotherapy efficacy is still controversial (32). In addition, studies have shown that the tumor microenvironment has a certain relationship with the response of patients receiving immunotherapy. TME immune cell infiltration has been considered as an important and inestimable information for predicting the prognosis of various cancers and immunotherapy responses (33, 34). In general, people have recently worked hard to develop markers that can identify patients who will benefit from immunotherapy, but there is no reliable marker that can be widely used in clinical practice. On these markers, our study explored the relationship between lncRNA markers and the immune function of BLCA patients. Differences in seven immune checkpoint genes including *PD-L1* gene between the two groups. Subsequently, the IMvigor data set patients receiving immunotherapy were divided into two groups according to their signatures. The low-risk group had a longer OS, indicating that the low-risk group was more likely to benefit from immunotherapy. Finally, we used the TIDE algorithm to predict the response of TCGA-BLCA patients to immunotherapy. In summary, our results indicate that patients in the low-risk group are more likely to benefit from immunotherapy. Unfortunately, due to the lack of independent samples of bladder cancer patients receiving immunotherapy, this conclusion cannot be verified in independent samples.

Recently, two studies have also constructed immune lncRNA signatures to predict the prognosis of bladder cancer patients. Compared with previous research, our research has certain advantages. First, we used frequency instead of predicting prognostic P value to screen out lncRNA. Secondly, compared with the Cao et al. study, our study has complete external verification and a unique cohort to verify our conclusions, which makes our conclusions more convincing (35). In addition, compared to the Song et al. study, our study explored the correlation between signatures and immunotherapy, and reached a more consistent conclusion (36). Finally, our signature has only four genes, so it is more cost-effective in future clinical applications.

It should be noted that our research has some limitations. First of all, this is a retrospective study. There may be a certain degree of heterogeneity among patients, and prospective studies are needed to verify our conclusions. Second, our research can only indirectly prove the relationship between the signature and the efficacy of immunotherapy in patients with bladder cancer.

## Conclusion

In Conclusion, the risk score signature based on four IRLs can effectively evaluate the prognosis of BLCA patients. In addition, the risk score has enormous implications for identifying ICI immunotherapy-sensitive patients.

## Data Availability Statement

Publicly available datasets were analyzed in this study. These data can be found here: https://cancergenome.nih.gov/.

## Author Contributions

LZ collected and analyzed the data and wrote the paper. LL contributed to experimental verification in the laboratory and participated in the writing. YZ assisted in the design of this study. ZZ is responsible for all the integrity of data and the accuracy of data analysis. All authors contributed to the article and approved the submitted version.

## Funding

The National Natural Science Foundation of China (No. 81702503) supported this study.

## Conflict of Interest

The authors declare that the research was conducted in the absence of any commercial or financial relationships that could be construed as a potential conflict of interest.
