# Supplementary material for: Identification of Immune-Related lncRNA Signature to Predict Prognosis and Immunotherapeutic Efficiency in Bladder Cancer
Source: Front Oncol. 2021 Jan 20;10:542140. doi: 10.3389/fonc.2020.542140 (PMC7855860; doi:10.3389/fonc.2020.542140)
Supplement: Supplementary file 1 [file DataSheet_1.docx]

**The process of screening robust lncRNA is as follows:**

library(survival)

library(glmnet)

library(pbapply)

library(survivalROC)

Sys.setenv(LANGUAGE = "en")

options(stringsAsFactors = FALSE)

display.progress = function (index, totalN, breakN=20) {

if ( index %% ceiling(totalN/breakN) ==0 ) {

cat(paste(round(index*100/totalN), "% ", sep=""))

}

}

surv_lasso <- function(iter.times = NULL, surv.obj = NULL, expr.obj = NULL, nfolds = 10, alpha = 1, family = "cox") {

cvfit = cv.glmnet(x = t(as.matrix(expr.obj)),

y = surv.obj,

nfolds = nfolds,

alpha = alpha,

family = family)

myCoefs <- coef(cvfit, s="lambda.min");

lasso_fea <- myCoefs@Dimnames[[1]][which(myCoefs != 0 )]

return(lasso_fea)

}

expr <- read.csv("easy_input_expr.csv", check.names = F, row.names = 1)

expr[1:3,1:3]

Sinfo <- read.csv("easy_input_cli.csv", check.names = F, row.names = 1)

Sinfo[1:3,1:3]

var.cutoff <- 0.2

var <- apply(expr, 1, sd)

expr.filtered <- expr[var > var.cutoff,]

cox.cutoff <- 0.5

Coxoutput.OS <- NULL

for (i in 1:nrow(expr.filtered)) {

display.progress(index = i,totalN = nrow(expr.filtered))

tmp <- data.frame(gene = as.numeric(expr.filtered[i,]),

OS.time = Sinfo[,"OS.time"],

OS = Sinfo[,"OS"],

stringsAsFactors = F)

cox <- coxph(Surv(OS.time, OS) ~ gene, data = tmp)

coxSummary = summary(cox)

Coxoutput.OS=rbind.data.frame(Coxoutput.OS,data.frame(gene=rownames(expr.filtered)[i],

HR=as.numeric(coxSummary$coefficients[,"exp(coef)"]),

z=as.numeric(coxSummary$coefficients[,"z"]),

pvalue=as.numeric(coxSummary$coefficients[,"Pr(>|z|)"]),

lower=as.numeric(coxSummary$conf.int[,3]),

upper=as.numeric(coxSummary$conf.int[,4]),

stringsAsFactors = F),

stringsAsFactors = F)

}

if(!identical(rownames(expr.filtered),Coxoutput.OS$gene)) {stop("Error! Gene name is mismatched!\n")}

surv.expr <- expr.filtered[Coxoutput.OS$pvalue < cox.cutoff,]

dim(surv.expr)

iter.times <- 1000

lasso_fea <- list()

surv <- Surv(Sinfo$OS.time, Sinfo$OS)

set.seed(111)

lasso_fea <- pblapply(1:iter.times,

surv_lasso,

surv.obj = surv,

expr.obj = surv.expr)

genes <- sort(table(unlist(lasso_fea)), decreasing = T)

freq.cutoff <- 500

genes <- names(genes[genes > freq.cutoff])

write.table(genes, "Robustgene.txt", row.names = F, quote = F)

**Signature construction**

pred.time <- 5

roc <- list()

auc <- c()

for (i in 1:length(genes)) {

gene <- genes[i]

tmp <- data.frame(gene = as.numeric(surv.expr[gene,]),row.names = colnames(surv.expr),stringsAsFactors = F); colnames(tmp) = gene

if(i == 1) {

surv.dat <- cbind.data.frame(Sinfo[rownames(tmp),c("OS.time","OS")],tmp)

} else {

surv.dat <- cbind.data.frame(surv.dat,tmp)

}

cox <- coxph(Surv(OS.time, OS) ~ ., data = surv.dat)

riskScore <- predict(cox,type="risk",newdata=surv.dat)

roc[[i]] <- survivalROC(Stime=surv.dat$OS.time,

status=surv.dat$OS,

marker = riskScore[rownames(surv.dat)],

predict.time =pred.time*365,

method="KM")

auc <- c(auc,roc[[i]]$AUC)

}

prog.sig <- genes[1:which.max(auc)]

write.table(prog.sig,"signature_gene.txt", row.names = F, quote = F)
