# Supplementary material for: Identification of Immune-Related lncRNA Signature to Predict Prognosis and Immunotherapeutic Efficiency in Bladder Cancer
Source: Front Oncol. 2021 Jan 20;10:542140. doi: 10.3389/fonc.2020.542140 (PMC7855860; doi:10.3389/fonc.2020.542140)
Supplement: Supplementary file 2 [file Table_1.docx]

| ID | F PRIMER | R PRIMER | PRODUCT SIZE |
| --- | --- | --- | --- |
| IPO5P1 | ATGGAAGGGATGAAGGCTCT | AGGCAGGAACATCAAACCAC | 194 |
| LINC00942 | CTGGCGTCTCTGATTTCCTC | CCATTTGGCCACTGAAGTCT | 128 |
| LINC01356 | CTCTGCTCCTCAGACCAACC | GGTCACAGGGGATATGATGG | 211 |
| HCP5 | GCTGGACGATTCTCCTCACACT | CTCCTCTCCAGGCACAGGTAAT | |

Table S1: The detailed primer sequences included in this study
