# Supplementary material for: Identification of Immune-Related lncRNA Signature to Predict Prognosis and Immunotherapeutic Efficiency in Bladder Cancer
Source: Front Oncol. 2021 Jan 20;10:542140. doi: 10.3389/fonc.2020.542140 (PMC7855860; doi:10.3389/fonc.2020.542140)
Supplement: Supplementary file 3 [file Table_2.docx]

|  | | | HCP5 | IPO5P1 | LINC00942 | LINC01356 | riskScore |
| --- | --- | --- | --- | --- | --- | --- | --- |
| TCGA-BL-A0C8 | | 2.181 | | 76.161 | 0.324 | 0.080 | -7.981 |
| TCGA-G2-AA3B | 25.241 | | | 21.609 | 0.975 | 0.017 | -2.907 |
| TCGA-ZF-A9R4 | 40.235 | | | 16.163 | 0.008 | 0.000 | -2.755 |
| TCGA-FJ-A3Z9 | 0.755 | | | 25.510 | 0.000 | 0.100 | -2.674 |
| TCGA-G2-A3IE | 1.503 | | | 23.426 | 0.219 | 0.020 | -2.475 |
| TCGA-GV-A6ZA | 39.839 | | | 13.566 | 0.046 | 0.000 | -2.474 |
| TCGA-ZF-A9R7 | 58.283 | | | 8.791 | 0.323 | 0.303 | -2.456 |
| TCGA-GC-A3I6 | 69.693 | | | 3.572 | 0.710 | 0.139 | -2.215 |
| TCGA-CF-A3MF | 0.647 | | | 20.372 | 0.089 | 0.000 | -2.137 |
| TCGA-E7-A3X6 | 75.958 | | | 1.055 | 0.049 | 0.032 | -2.133 |
| TCGA-FD-A43P | 47.645 | | | 7.552 | 0.084 | 0.129 | -2.052 |
| TCGA-2F-A9KO | 65.848 | | | 2.393 | 0.076 | 0.043 | -2.002 |
| TCGA-K4-A5RH | 69.107 | | | 1.568 | 0.087 | 0.578 | -1.990 |
| TCGA-UY-A9PB | 63.552 | | | 2.826 | 0.099 | 1.433 | -1.952 |
| TCGA-2F-A9KR | 23.814 | | | 12.760 | 1.141 | 0.000 | -1.946 |
| TCGA-E7-A6ME | 43.537 | | | 7.420 | 0.084 | 0.042 | -1.931 |
| TCGA-CF-A27C | 8.118 | | | 16.401 | 0.042 | 0.000 | -1.924 |
| TCGA-DK-A6B6 | 26.862 | | | 11.440 | 0.033 | 0.044 | -1.905 |
| TCGA-UY-A9PH | 52.264 | | | 4.939 | 0.149 | 0.020 | -1.904 |
| TCGA-ZF-A9RL | 5.368 | | | 16.746 | 0.021 | 0.056 | -1.885 |
| TCGA-CU-A3YL | 13.011 | | | 14.206 | 0.068 | 0.034 | -1.824 |
| TCGA-XF-AAML | 36.901 | | | 7.854 | 0.051 | 0.017 | -1.800 |
| TCGA-GC-A3WC | 57.849 | | | 3.157 | 5.493 | 0.059 | -1.786 |
| TCGA-ZF-A9R5 | 24.638 | | | 10.830 | 0.037 | 0.073 | -1.782 |
| TCGA-E7-A8O7 | 16.086 | | | 12.771 | 0.090 | 0.018 | -1.757 |
| TCGA-LC-A66R | 58.018 | | | 2.162 | 0.075 | 0.851 | -1.750 |
| TCGA-XF-A9SM | 57.966 | | | 1.860 | 0.302 | 0.297 | -1.727 |
| TCGA-E5-A4U1 | 2.306 | | | 15.978 | 0.041 | 0.014 | -1.724 |
| TCGA-XF-AAMX | 1.264 | | | 16.102 | 0.130 | 0.000 | -1.708 |
| TCGA-K4-A5RJ | 55.915 | | | 2.137 | 0.034 | 0.240 | -1.706 |
| TCGA-E7-A677 | 12.171 | | | 13.327 | 0.192 | 0.601 | -1.695 |
| TCGA-K4-A3WS | 27.020 | | | 9.064 | 0.062 | 0.035 | -1.662 |
| TCGA-XF-A8HD | 57.836 | | | 1.697 | 0.082 | 2.424 | -1.658 |
| TCGA-FJ-A3ZF | 0.838 | | | 15.637 | 0.089 | 0.022 | -1.649 |
| TCGA-FD-A3B6 | 49.000 | | | 3.313 | 0.078 | 0.412 | -1.640 |
| TCGA-GU-A766 | 59.614 | | | 0.580 | 0.095 | 0.491 | -1.636 |
| TCGA-E7-A6MF | 7.011 | | | 13.767 | 0.701 | 0.083 | -1.608 |
| TCGA-YF-AA3L | 1.887 | | | 14.974 | 0.445 | 0.025 | -1.602 |
| TCGA-G2-AA3F | 1.145 | | | 15.088 | 0.033 | 0.022 | -1.600 |
| TCGA-GU-A762 | 55.471 | | | 1.373 | 1.003 | 0.901 | -1.584 |
| TCGA-E7-A7XN | 59.198 | | | 0.121 | 0.009 | 0.251 | -1.584 |
| TCGA-PQ-A6FI | 55.954 | | | 0.754 | 0.044 | 0.117 | -1.566 |
| TCGA-ZF-AA4X | 5.998 | | | 13.367 | 0.261 | 0.000 | -1.548 |
| TCGA-GD-A76B | 42.461 | | | 4.042 | 0.081 | 0.146 | -1.548 |
| TCGA-GV-A3JX | 42.737 | | | 3.846 | 0.132 | 0.108 | -1.535 |
| TCGA-GV-A3JW | 0.840 | | | 14.283 | 0.314 | 0.009 | -1.505 |
| TCGA-G2-A2EK | 4.073 | | | 13.348 | 0.208 | 0.000 | -1.495 |
| TCGA-CF-A7I0 | 2.473 | | | 13.717 | 0.019 | 0.000 | -1.494 |
| TCGA-KQ-A41O | 0.736 | | | 14.049 | 0.049 | 0.000 | -1.482 |
| TCGA-CF-A9FH | 22.176 | | | 8.470 | 0.022 | 0.044 | -1.472 |
| TCGA-DK-A3WW | 54.837 | | | 0.121 | 0.177 | 0.160 | -1.468 |
| TCGA-XF-AAMZ | 2.342 | | | 13.428 | 0.016 | 0.000 | -1.460 |
| TCGA-G2-A2EJ | 52.398 | | | 0.418 | 0.038 | 0.109 | -1.437 |
| TCGA-E7-A678 | 13.914 | | | 10.141 | 0.070 | 0.000 | -1.426 |
| TCGA-4Z-AA7M | 2.016 | | | 13.194 | 0.106 | 0.014 | -1.426 |
| TCGA-ZF-A9RM | 1.738 | | | 12.915 | 0.051 | 0.011 | -1.390 |
| TCGA-ZF-AA58 | 49.106 | | | 1.049 | 0.089 | 1.275 | -1.386 |
| TCGA-FD-A3B4 | 48.788 | | | 0.957 | 0.082 | 0.832 | -1.379 |
| TCGA-DK-AA6W | 3.613 | | | 12.251 | 0.447 | 0.221 | -1.360 |
| TCGA-S5-AA26 | 0.315 | | | 12.930 | 0.012 | 0.016 | -1.354 |
| TCGA-CF-A47V | 13.512 | | | 9.525 | 0.016 | 0.031 | -1.351 |
| TCGA-BT-A3PJ | 47.260 | | | 1.112 | 0.337 | 0.800 | -1.351 |
| TCGA-GV-A3QI | 21.025 | | | 7.573 | 0.055 | 0.049 | -1.347 |
| TCGA-ZF-A9RF | 34.106 | | | 4.387 | 0.026 | 0.807 | -1.346 |
| TCGA-ZF-A9RC | 0.914 | | | 12.729 | 0.184 | 0.214 | -1.342 |
| TCGA-XF-AAMY | 1.306 | | | 12.497 | 0.029 | 0.252 | -1.329 |
| TCGA-FT-A61P | 47.078 | | | 0.694 | 0.009 | 0.171 | -1.323 |
| TCGA-XF-AAN1 | 7.986 | | | 10.501 | 0.000 | 0.018 | -1.306 |
| TCGA-DK-A3WY | 35.676 | | | 3.452 | 0.075 | 0.264 | -1.303 |
| TCGA-4Z-AA89 | 31.643 | | | 4.295 | 0.055 | 0.043 | -1.289 |
| TCGA-DK-A3IK | 4.579 | | | 11.058 | 0.020 | 0.090 | -1.271 |
| TCGA-GC-A3RB | 28.990 | | | 4.523 | 0.038 | 0.058 | -1.242 |
| TCGA-BT-A2LA | 14.174 | | | 8.957 | 0.041 | 2.973 | -1.238 |
| TCGA-XF-A8HC | 3.611 | | | 10.966 | 0.000 | 0.030 | -1.237 |
| TCGA-UY-A9PA | 37.279 | | | 2.316 | 0.179 | 0.206 | -1.227 |
| TCGA-UY-A78P | 41.461 | | | 1.126 | 0.027 | 0.166 | -1.218 |
| TCGA-LT-A8JT | 11.556 | | | 8.688 | 0.008 | 0.030 | -1.212 |
| TCGA-GU-AATP | 23.094 | | | 5.723 | 0.025 | 0.065 | -1.209 |
| TCGA-G2-AA3D | 3.442 | | | 10.738 | 0.097 | 0.077 | -1.207 |
| TCGA-4Z-AA7O | 32.621 | | | 3.222 | 0.063 | 0.017 | -1.204 |
| TCGA-CF-A47S | 2.612 | | | 10.902 | 0.100 | 0.011 | -1.203 |
| TCGA-UY-A9PD | 10.338 | | | 8.906 | 0.042 | 0.018 | -1.202 |
| TCGA-G2-A3VY | 2.630 | | | 10.792 | 0.040 | 0.018 | -1.193 |
| TCGA-DK-A3IV | 26.638 | | | 4.612 | 0.223 | 0.008 | -1.187 |
| TCGA-K4-A3WV | 14.930 | | | 7.513 | 0.072 | 0.250 | -1.173 |
| TCGA-GC-A3BM | 16.043 | | | 7.149 | 0.138 | 0.043 | -1.169 |
| TCGA-ZF-A9R2 | 3.042 | | | 10.405 | 0.148 | 0.000 | -1.162 |
| TCGA-GV-A3JV | 8.572 | | | 8.964 | 0.053 | 0.026 | -1.160 |
| TCGA-FD-A43X | 3.841 | | | 10.167 | 0.530 | 0.009 | -1.153 |
| TCGA-GV-A3QK | 14.284 | | | 7.327 | 0.030 | 0.024 | -1.143 |
| TCGA-XF-A9T5 | 38.909 | | | 1.037 | 0.310 | 0.026 | -1.140 |
| TCGA-4Z-AA7W | 40.108 | | | 0.823 | 0.043 | 0.730 | -1.136 |
| TCGA-DK-A3IS | 31.523 | | | 2.690 | 0.006 | 0.033 | -1.119 |
| TCGA-FD-A5BR | 9.850 | | | 8.184 | 0.119 | 0.132 | -1.110 |
| TCGA-LT-A5Z6 | 5.564 | | | 9.236 | 0.041 | 0.000 | -1.109 |
| TCGA-FD-A43S | 9.949 | | | 8.058 | 0.156 | 0.092 | -1.100 |
| TCGA-FD-A43Y | 39.863 | | | 0.521 | 1.148 | 0.171 | -1.095 |
| TCGA-FD-A6TE | 29.823 | | | 2.868 | 0.194 | 0.008 | -1.090 |
| TCGA-G2-A2EL | 24.258 | | | 4.344 | 0.031 | 0.497 | -1.086 |
| TCGA-ZF-A9R0 | 15.628 | | | 6.430 | 0.123 | 0.000 | -1.084 |
| TCGA-UY-A78K | 11.086 | | | 7.504 | 0.016 | 0.010 | -1.076 |
| TCGA-FJ-A3ZE | 3.038 | | | 9.574 | 0.088 | 0.029 | -1.076 |
| TCGA-FD-A3N6 | 41.451 | | | 0.020 | 2.123 | 0.016 | -1.074 |
| TCGA-XF-AAMG | 8.808 | | | 8.164 | 0.039 | 0.522 | -1.071 |
| TCGA-DK-A2I4 | 37.846 | | | 0.659 | 0.042 | 0.311 | -1.069 |
| TCGA-GU-A763 | 11.230 | | | 7.261 | 0.024 | 0.032 | -1.054 |
| TCGA-XF-AAN5 | 34.822 | | | 1.158 | 0.243 | 0.347 | -1.037 |
| TCGA-FD-A5C1 | 36.438 | | | 0.754 | 0.294 | 0.496 | -1.033 |
| TCGA-DK-A6AW | 3.536 | | | 8.982 | 0.068 | 0.103 | -1.026 |
| TCGA-E7-A4XJ | 0.719 | | | 9.903 | 0.118 | 1.050 | -1.023 |
| TCGA-DK-AA6X | 23.239 | | | 4.009 | 0.885 | 0.084 | -1.021 |
| TCGA-FD-A3N5 | 32.283 | | | 1.392 | 0.012 | 0.100 | -1.003 |
| TCGA-UY-A8OB | 35.751 | | | 0.820 | 0.231 | 1.483 | -0.999 |
| TCGA-G2-A2EC | 6.567 | | | 7.909 | 0.057 | 0.009 | -0.997 |
| TCGA-4Z-AA87 | 1.679 | | | 9.148 | 0.000 | 0.010 | -0.997 |
| TCGA-FD-A3B8 | 33.952 | | | 0.868 | 0.070 | 0.129 | -0.991 |
| TCGA-DK-AA76 | 0.573 | | | 9.204 | 0.052 | 0.000 | -0.973 |
| TCGA-4Z-AA86 | 34.299 | | | 0.870 | 0.776 | 0.912 | -0.971 |
| TCGA-DK-AA6L | 21.845 | | | 3.797 | 0.298 | 0.102 | -0.971 |
| TCGA-ZF-A9RD | 35.683 | | | 0.193 | 0.082 | 0.087 | -0.968 |
| TCGA-ZF-AA4R | 28.179 | | | 2.156 | 0.418 | 0.108 | -0.967 |
| TCGA-E7-A97Q | 18.014 | | | 4.653 | 0.064 | 0.024 | -0.963 |
| TCGA-E7-A85H | 5.542 | | | 7.760 | 0.105 | 0.010 | -0.954 |
| TCGA-XF-A9T4 | 33.917 | | | 0.520 | 0.284 | 0.042 | -0.953 |
| TCGA-FD-A3SR | 20.539 | | | 3.888 | 0.092 | 0.066 | -0.949 |
| TCGA-DK-AA6U | 5.051 | | | 7.734 | 0.143 | 0.120 | -0.935 |
| TCGA-GV-A3QH | 9.713 | | | 6.483 | 0.006 | 0.033 | -0.933 |
| TCGA-C4-A0F1 | 34.806 | | | 0.032 | 0.015 | 0.010 | -0.931 |
| TCGA-DK-A1A6 | 2.344 | | | 8.293 | 0.013 | 0.009 | -0.926 |
| TCGA-4Z-AA83 | 12.005 | | | 5.729 | 0.060 | 0.016 | -0.915 |
| TCGA-FD-A3SQ | 16.056 | | | 4.701 | 0.074 | 0.065 | -0.915 |
| TCGA-4Z-AA7N | 23.651 | | | 2.728 | 0.085 | 0.023 | -0.913 |
| TCGA-HQ-A5ND | 33.717 | | | 0.127 | 0.000 | 0.000 | -0.912 |
| TCGA-4Z-AA7Q | 19.795 | | | 3.672 | 0.067 | 0.148 | -0.905 |
| TCGA-DK-AA71 | 12.457 | | | 5.475 | 0.045 | 0.015 | -0.901 |
| TCGA-BT-A20W | 8.051 | | | 6.622 | 0.122 | 0.081 | -0.900 |
| TCGA-FD-A5BY | 28.718 | | | 1.391 | 0.882 | 0.136 | -0.894 |
| TCGA-E7-A97P | 28.903 | | | 1.170 | 0.091 | 0.206 | -0.886 |
| TCGA-E7-A3Y1 | 4.916 | | | 7.257 | 0.045 | 0.012 | -0.886 |
| TCGA-FD-A3B7 | 28.903 | | | 1.317 | 0.137 | 0.893 | -0.884 |
| TCGA-FD-A62N | 26.453 | | | 1.679 | 0.383 | 0.037 | -0.873 |
| TCGA-ZF-A9R3 | 11.309 | | | 5.513 | 0.122 | 0.040 | -0.873 |
| TCGA-YC-A8S6 | 6.626 | | | 6.694 | 0.039 | 0.051 | -0.872 |
| TCGA-BT-A2LB | 26.934 | | | 1.584 | 0.786 | 0.000 | -0.871 |
| TCGA-GU-A42P | 0.891 | | | 8.346 | 0.006 | 1.080 | -0.867 |
| TCGA-DK-AA74 | 27.892 | | | 1.239 | 0.137 | 0.404 | -0.860 |
| TCGA-CF-A5U8 | 3.759 | | | 7.344 | 0.243 | 0.037 | -0.860 |
| TCGA-HQ-A2OF | 4.852 | | | 7.277 | 0.076 | 1.076 | -0.860 |
| TCGA-GV-A3QF | 0.490 | | | 8.124 | 0.026 | 0.000 | -0.859 |
| TCGA-DK-A1AG | 9.730 | | | 5.760 | 0.037 | 0.030 | -0.858 |
| TCGA-FD-A6TB | 18.162 | | | 3.766 | 0.450 | 0.784 | -0.850 |
| TCGA-BT-A20T | 24.778 | | | 1.857 | 0.277 | 0.000 | -0.850 |
| TCGA-DK-A6B1 | 6.986 | | | 6.371 | 0.020 | 0.009 | -0.849 |
| TCGA-XF-A9T8 | 31.387 | | | 0.103 | 0.065 | 0.011 | -0.846 |
| TCGA-XF-A9SI | 24.765 | | | 1.832 | 0.142 | 0.126 | -0.846 |
| TCGA-FD-A62O | 0.934 | | | 7.917 | 0.142 | 0.075 | -0.845 |
| TCGA-XF-A9SK | 19.688 | | | 3.144 | 0.300 | 0.327 | -0.840 |
| TCGA-XF-A9T3 | 24.447 | | | 1.858 | 0.254 | 0.084 | -0.839 |
| TCGA-E7-A4IJ | 11.228 | | | 5.212 | 0.472 | 0.028 | -0.834 |
| TCGA-CU-A3KJ | 24.753 | | | 1.670 | 0.020 | 0.040 | -0.832 |
| TCGA-GD-A3OP | 5.507 | | | 6.599 | 0.054 | 0.056 | -0.832 |
| TCGA-DK-A3IU | 21.107 | | | 2.616 | 0.013 | 0.689 | -0.818 |
| TCGA-E7-A7PW | 3.579 | | | 6.968 | 0.145 | 0.074 | -0.817 |
| TCGA-K4-A6FZ | 24.456 | | | 1.592 | 0.319 | 0.000 | -0.813 |
| TCGA-FD-A3NA | 18.205 | | | 3.111 | 0.073 | 0.123 | -0.805 |
| TCGA-XF-AAN2 | 23.826 | | | 1.636 | 0.185 | 0.076 | -0.801 |
| TCGA-4Z-AA81 | 27.987 | | | 0.581 | 0.053 | 0.251 | -0.800 |
| TCGA-XF-AAMQ | 15.856 | | | 3.582 | 0.099 | 0.048 | -0.793 |
| TCGA-KQ-A41R | 1.404 | | | 7.261 | 0.013 | 0.018 | -0.793 |
| TCGA-H4-A2HO | 2.632 | | | 6.845 | 0.163 | 0.036 | -0.779 |
| TCGA-E7-A541 | 14.895 | | | 3.659 | 0.139 | 0.040 | -0.775 |
| TCGA-DK-A6B5 | 23.625 | | | 1.480 | 0.122 | 0.487 | -0.770 |
| TCGA-DK-AA77 | 20.363 | | | 2.105 | 0.009 | 0.025 | -0.761 |
| TCGA-BT-A20O | 32.835 | | | 1.163 | 15.667 | 0.191 | -0.754 |
| TCGA-UY-A78N | 0.713 | | | 6.949 | 0.022 | 0.019 | -0.742 |
| TCGA-FD-A5BV | 1.008 | | | 6.872 | 0.118 | 0.104 | -0.738 |
| TCGA-XF-A8HF | 2.528 | | | 6.424 | 0.027 | 0.012 | -0.736 |
| TCGA-SY-A9G0 | 4.571 | | | 5.889 | 0.073 | 0.048 | -0.733 |
| TCGA-FD-A5BS | 23.786 | | | 1.572 | 4.450 | 0.105 | -0.728 |
| TCGA-ZF-AA4U | 11.812 | | | 3.960 | 0.020 | 0.000 | -0.727 |
| TCGA-GV-A40G | 1.644 | | | 6.552 | 0.040 | 0.009 | -0.725 |
| TCGA-FD-A6TD | 25.114 | | | 0.831 | 0.478 | 1.033 | -0.724 |
| TCGA-E7-A519 | 9.699 | | | 4.573 | 1.103 | 0.035 | -0.717 |
| TCGA-K4-A3WU | 10.008 | | | 4.448 | 0.404 | 0.390 | -0.714 |
| TCGA-XF-A8HB | 9.134 | | | 4.530 | 0.021 | 0.065 | -0.713 |
| TCGA-ZF-AA4W | 25.794 | | | 0.380 | 0.000 | 0.644 | -0.711 |
| TCGA-CF-A1HR | 8.657 | | | 4.613 | 0.066 | 0.014 | -0.710 |
| TCGA-H4-A2HQ | 10.520 | | | 4.104 | 0.054 | 0.000 | -0.707 |
| TCGA-XF-A9SU | 2.177 | | | 6.210 | 0.131 | 0.047 | -0.701 |
| TCGA-DK-AA6M | 19.633 | | | 2.017 | 1.418 | 1.008 | -0.687 |
| TCGA-FD-A3SP | 18.356 | | | 2.086 | 0.107 | 0.737 | -0.687 |
| TCGA-BT-A20Q | 20.957 | | | 1.213 | 0.089 | 0.024 | -0.683 |
| TCGA-K4-A83P | 15.469 | | | 2.617 | 0.071 | 0.042 | -0.683 |
| TCGA-DK-A6B2 | 12.964 | | | 3.348 | 0.168 | 0.584 | -0.677 |
| TCGA-DK-AA6S | 15.278 | | | 2.631 | 0.344 | 0.000 | -0.676 |
| TCGA-CF-A47W | 3.289 | | | 5.627 | 0.050 | 0.019 | -0.672 |
| TCGA-ZF-AA51 | 10.457 | | | 3.811 | 0.078 | 0.086 | -0.672 |
| TCGA-XF-A8HI | 10.582 | | | 3.716 | 0.104 | 0.025 | -0.667 |
| TCGA-G2-A2EF | 11.995 | | | 3.947 | 0.127 | 2.562 | -0.667 |
| TCGA-E5-A4TZ | 3.248 | | | 5.591 | 0.032 | 0.093 | -0.666 |
| TCGA-FD-A3B3 | 20.881 | | | 1.092 | 0.027 | 0.169 | -0.666 |
| TCGA-GC-A3RC | 19.890 | | | 1.391 | 0.316 | 0.319 | -0.662 |
| TCGA-DK-A6B0 | 4.310 | | | 5.228 | 0.224 | 0.010 | -0.656 |
| TCGA-KQ-A41P | 7.608 | | | 4.423 | 0.277 | 0.178 | -0.655 |
| TCGA-ZF-AA53 | 23.634 | | | 0.399 | 0.081 | 0.663 | -0.654 |
| TCGA-CF-A47X | 4.198 | | | 5.184 | 0.024 | 0.000 | -0.651 |
| TCGA-K4-AAQO | 6.051 | | | 4.709 | 0.100 | 0.013 | -0.650 |
| TCGA-GD-A3OS | 23.002 | | | 0.519 | 0.114 | 0.731 | -0.648 |
| TCGA-DK-A3WX | 22.747 | | | 0.477 | 0.177 | 0.418 | -0.643 |
| TCGA-HQ-A5NE | 19.294 | | | 1.323 | 0.386 | 0.190 | -0.641 |
| TCGA-G2-A2ES | 22.776 | | | 0.324 | 0.026 | 0.026 | -0.640 |
| TCGA-GU-A767 | 3.555 | | | 5.176 | 0.026 | 0.000 | -0.633 |
| TCGA-CU-A0YO | 14.880 | | | 2.293 | 0.169 | 0.025 | -0.632 |
| TCGA-XF-A9SP | 4.414 | | | 5.055 | 0.159 | 0.487 | -0.630 |
| TCGA-C4-A0EZ | 1.356 | | | 6.274 | 0.047 | 2.676 | -0.624 |
| TCGA-4Z-AA7Y | 6.647 | | | 4.287 | 0.019 | 0.025 | -0.623 |
| TCGA-FD-A6TA | 8.019 | | | 3.935 | 0.010 | 0.027 | -0.623 |
| TCGA-5N-A9KM | 5.448 | | | 4.466 | 0.000 | 0.061 | -0.609 |
| TCGA-XF-A9SX | 15.524 | | | 2.105 | 0.695 | 0.585 | -0.608 |
| TCGA-DK-A1AB | 18.463 | | | 1.122 | 0.050 | 0.051 | -0.607 |
| TCGA-SY-A9G5 | 18.752 | | | 1.207 | 0.700 | 0.745 | -0.597 |
| TCGA-XF-AAMT | 18.075 | | | 1.271 | 0.664 | 0.320 | -0.596 |
| TCGA-XF-AAMR | 13.690 | | | 2.208 | 0.016 | 0.058 | -0.593 |
| TCGA-DK-AA6T | 8.143 | | | 3.617 | 0.030 | 0.119 | -0.590 |
| TCGA-FD-A6TK | 19.811 | | | 0.817 | 0.398 | 0.707 | -0.590 |
| TCGA-FJ-A3Z7 | 5.567 | | | 4.230 | 0.008 | 0.020 | -0.588 |
| TCGA-CF-A47Y | 6.708 | | | 3.934 | 0.502 | 0.010 | -0.581 |
| TCGA-BT-A20P | 9.154 | | | 3.220 | 0.105 | 0.000 | -0.578 |
| TCGA-5N-A9KI | 15.357 | | | 1.646 | 0.181 | 0.033 | -0.577 |
| TCGA-BL-A3JM | 11.501 | | | 2.614 | 0.134 | 0.039 | -0.576 |
| TCGA-XF-AAMH | 1.120 | | | 5.322 | 0.103 | 0.399 | -0.573 |
| TCGA-FD-A3SO | 17.936 | | | 1.076 | 0.298 | 0.620 | -0.570 |
| TCGA-XF-A9SJ | 15.997 | | | 1.448 | 0.037 | 0.343 | -0.568 |
| TCGA-CF-A8HX | 1.209 | | | 5.082 | 0.671 | 0.024 | -0.551 |
| TCGA-DK-A1A5 | 19.431 | | | 0.353 | 0.199 | 0.056 | -0.550 |
| TCGA-2F-A9KP | 1.025 | | | 5.035 | 0.058 | 0.033 | -0.550 |
| TCGA-ZF-AA5H | 17.144 | | | 1.253 | 0.647 | 1.223 | -0.548 |
| TCGA-UY-A78O | 1.646 | | | 4.752 | 0.047 | 0.000 | -0.538 |
| TCGA-XF-AAME | 13.181 | | | 1.832 | 0.054 | 0.246 | -0.535 |
| TCGA-CU-A5W6 | 2.552 | | | 4.501 | 0.133 | 0.082 | -0.533 |
| TCGA-XF-A9SY | 19.337 | | | 0.284 | 0.158 | 0.554 | -0.529 |
| TCGA-FD-A3B5 | 17.543 | | | 0.684 | 0.386 | 0.198 | -0.528 |
| TCGA-CF-A9FF | 4.126 | | | 3.992 | 0.049 | 0.033 | -0.524 |
| TCGA-GC-A3YS | 14.279 | | | 1.550 | 0.105 | 0.799 | -0.521 |
| TCGA-CF-A9FM | 5.996 | | | 3.504 | 0.269 | 0.013 | -0.520 |
| TCGA-DK-A3IN | 6.128 | | | 3.843 | 0.222 | 1.760 | -0.517 |
| TCGA-GU-A42R | 1.650 | | | 4.537 | 0.227 | 0.011 | -0.513 |
| TCGA-GV-A3JZ | 3.521 | | | 3.997 | 0.024 | 0.039 | -0.509 |
| TCGA-DK-AA6P | 1.806 | | | 4.420 | 0.022 | 0.000 | -0.508 |
| TCGA-ZF-AA4T | 5.700 | | | 3.428 | 0.000 | 0.069 | -0.507 |
| TCGA-DK-A1AD | 8.520 | | | 2.688 | 0.046 | 0.009 | -0.506 |
| TCGA-ZF-AA5P | 11.177 | | | 2.014 | 0.029 | 0.051 | -0.506 |
| TCGA-ZF-A9RN | 14.921 | | | 1.001 | 0.126 | 0.064 | -0.498 |
| TCGA-S5-A6DX | 4.581 | | | 3.964 | 0.170 | 1.442 | -0.497 |
| TCGA-GC-A3OO | 4.076 | | | 3.796 | 0.440 | 0.047 | -0.496 |
| TCGA-XF-A8HH | 2.053 | | | 4.248 | 0.188 | 0.026 | -0.494 |
| TCGA-4Z-AA82 | 13.870 | | | 1.289 | 0.103 | 0.383 | -0.493 |
| TCGA-XF-AAN0 | 7.393 | | | 2.951 | 0.723 | 0.051 | -0.492 |
| TCGA-CF-A3MI | 1.686 | | | 4.253 | 0.008 | 0.000 | -0.488 |
| TCGA-FD-A3SJ | 3.194 | | | 3.869 | 0.151 | 0.008 | -0.486 |
| TCGA-DK-A2I1 | 4.909 | | | 3.410 | 0.000 | 0.019 | -0.485 |
| TCGA-R3-A69X | 11.091 | | | 1.807 | 0.016 | 0.000 | -0.484 |
| TCGA-BT-A42E | 16.009 | | | 0.673 | 0.028 | 0.643 | -0.481 |
| TCGA-YC-A89H | 1.453 | | | 4.227 | 0.009 | 0.023 | -0.478 |
| TCGA-CF-A3MH | 1.122 | | | 4.260 | 0.056 | 0.018 | -0.472 |
| TCGA-GD-A6C6 | 4.801 | | | 3.329 | 0.180 | 0.000 | -0.472 |
| TCGA-GC-A3RD | 3.642 | | | 3.610 | 0.009 | 0.045 | -0.472 |
| TCGA-CF-A47T | 0.625 | | | 4.367 | 0.036 | 0.000 | -0.471 |
| TCGA-BT-A42C | 5.262 | | | 3.169 | 0.000 | 0.128 | -0.467 |
| TCGA-FD-A3SS | 2.793 | | | 3.917 | 0.095 | 0.622 | -0.466 |
| TCGA-KQ-A41Q | 2.846 | | | 3.765 | 0.113 | 0.025 | -0.466 |
| TCGA-HQ-A2OE | 1.719 | | | 4.090 | 0.107 | 0.198 | -0.465 |
| TCGA-ZF-AA54 | 14.461 | | | 0.825 | 0.111 | 0.213 | -0.464 |
| TCGA-E5-A2PC | 46.002 | | | 0.564 | 54.120 | 0.053 | -0.464 |
| TCGA-E7-A6MD | 6.252 | | | 2.862 | 0.039 | 0.026 | -0.463 |
| TCGA-4Z-AA7S | 1.030 | | | 4.195 | 0.000 | 0.083 | -0.462 |
| TCGA-K4-A4AC | 17.631 | | | 0.370 | 0.079 | 1.900 | -0.461 |
| TCGA-FD-A43N | 4.084 | | | 3.409 | 0.046 | 0.148 | -0.459 |
| TCGA-DK-A1AF | 13.232 | | | 0.986 | 0.042 | 0.016 | -0.454 |
| TCGA-XF-A9SL | 7.308 | | | 2.418 | 0.047 | 0.037 | -0.445 |
| TCGA-XF-AAMJ | 7.048 | | | 2.465 | 0.038 | 0.017 | -0.444 |
| TCGA-FD-A5BX | 15.075 | | | 0.404 | 0.031 | 0.177 | -0.439 |
| TCGA-MV-A51V | 1.386 | | | 3.857 | 0.031 | 0.041 | -0.437 |
| TCGA-2F-A9KQ | 0.775 | | | 3.967 | 0.009 | 0.011 | -0.433 |
| TCGA-UY-A9PE | 1.639 | | | 3.916 | 0.141 | 0.664 | -0.433 |
| TCGA-XF-A9T6 | 13.273 | | | 1.185 | 0.633 | 1.457 | -0.432 |
| TCGA-CF-A9FL | 2.023 | | | 3.623 | 0.056 | 0.062 | -0.429 |
| TCGA-CF-A1HS | 13.560 | | | 0.700 | 0.064 | 0.199 | -0.428 |
| TCGA-GU-A764 | 3.929 | | | 3.508 | 1.491 | 0.884 | -0.426 |
| TCGA-K4-A6MB | 1.965 | | | 3.599 | 0.069 | 0.064 | -0.424 |
| TCGA-XF-A8HG | 0.224 | | | 4.021 | 0.019 | 0.039 | -0.423 |
| TCGA-XF-AAN7 | 5.355 | | | 2.703 | 0.053 | 0.000 | -0.423 |
| TCGA-4Z-AA7R | 3.388 | | | 3.205 | 0.134 | 0.024 | -0.421 |
| TCGA-DK-AA75 | 1.713 | | | 3.617 | 0.079 | 0.021 | -0.421 |
| TCGA-KQ-A41N | 0.574 | | | 3.934 | 0.086 | 0.456 | -0.413 |
| TCGA-CU-A0YR | 4.724 | | | 2.810 | 0.100 | 0.185 | -0.412 |
| TCGA-2F-A9KT | 1.010 | | | 3.710 | 0.118 | 0.000 | -0.411 |
| TCGA-GD-A3OQ | 11.182 | | | 1.144 | 0.374 | 0.016 | -0.411 |
| TCGA-ZF-A9RE | 6.667 | | | 2.201 | 0.021 | 0.027 | -0.406 |
| TCGA-ZF-A9R9 | 1.709 | | | 3.496 | 0.289 | 0.070 | -0.403 |
| TCGA-CU-A3QU | 7.636 | | | 1.822 | 0.027 | 0.009 | -0.393 |
| TCGA-DK-AA6Q | 52.008 | | | 0.677 | 70.152 | 0.100 | -0.392 |
| TCGA-UY-A78L | 2.628 | | | 3.216 | 0.036 | 0.590 | -0.390 |
| TCGA-FD-A6TI | 10.675 | | | 1.102 | 0.115 | 0.339 | -0.389 |
| TCGA-XF-A9SH | 1.425 | | | 3.377 | 0.227 | 0.020 | -0.386 |
| TCGA-DK-A3IM | 8.489 | | | 1.625 | 0.229 | 0.262 | -0.386 |
| TCGA-BT-A20X | 13.549 | | | 0.223 | 0.025 | 0.017 | -0.383 |
| TCGA-CU-A0YN | 9.418 | | | 1.448 | 0.356 | 0.750 | -0.378 |
| TCGA-FD-A43U | 7.112 | | | 1.858 | 0.408 | 0.082 | -0.375 |
| TCGA-DK-A1AC | 4.656 | | | 2.417 | 0.000 | 0.070 | -0.374 |
| TCGA-GV-A40E | 10.007 | | | 1.293 | 0.255 | 1.024 | -0.373 |
| TCGA-GD-A2C5 | 4.512 | | | 2.403 | 0.067 | 0.010 | -0.369 |
| TCGA-FD-A6TC | 2.201 | | | 3.089 | 0.120 | 0.424 | -0.368 |
| TCGA-GU-AATO | 5.392 | | | 2.209 | 0.444 | 0.000 | -0.367 |
| TCGA-DK-A2I6 | 10.814 | | | 1.047 | 0.138 | 1.285 | -0.364 |
| TCGA-XF-AAMW | 11.774 | | | 0.495 | 0.274 | 0.000 | -0.361 |
| TCGA-BL-A5ZZ | 4.996 | | | 2.357 | 0.008 | 0.727 | -0.361 |
| TCGA-ZF-AA52 | 5.379 | | | 2.098 | 0.022 | 0.030 | -0.361 |
| TCGA-DK-A2I2 | 11.883 | | | 0.421 | 0.049 | 0.183 | -0.355 |
| TCGA-BT-A3PK | 10.247 | | | 0.859 | 0.076 | 0.292 | -0.354 |
| TCGA-FD-A3SN | 8.050 | | | 1.551 | 0.478 | 0.645 | -0.353 |
| TCGA-ZF-AA5N | 2.851 | | | 3.099 | 0.022 | 1.866 | -0.353 |
| TCGA-XF-A9SW | 4.621 | | | 2.212 | 0.000 | 0.024 | -0.353 |
| TCGA-CF-A3MG | 0.661 | | | 3.152 | 0.010 | 0.000 | -0.346 |
| TCGA-XF-A9SZ | 2.145 | | | 2.773 | 0.053 | 0.014 | -0.345 |
| TCGA-FD-A6TH | 3.880 | | | 2.565 | 0.115 | 1.065 | -0.343 |
| TCGA-FD-A62S | 10.851 | | | 0.679 | 0.631 | 0.309 | -0.343 |
| TCGA-XF-A9T0 | 2.475 | | | 2.621 | 0.031 | 0.014 | -0.338 |
| TCGA-BT-A2LD | 5.650 | | | 2.137 | 0.000 | 1.454 | -0.338 |
| TCGA-DK-A6AV | 2.514 | | | 2.534 | 0.036 | 0.031 | -0.330 |
| TCGA-C4-A0F7 | 3.847 | | | 2.200 | 0.005 | 0.138 | -0.328 |
| TCGA-UY-A9PF | 2.669 | | | 2.461 | 0.045 | 0.060 | -0.325 |
| TCGA-C4-A0F6 | 4.122 | | | 2.072 | 0.042 | 0.000 | -0.325 |
| TCGA-CF-A8HY | 1.280 | | | 2.808 | 0.090 | 0.053 | -0.324 |
| TCGA-XF-A8HE | 9.800 | | | 0.551 | 0.000 | 0.034 | -0.318 |
| TCGA-CF-A5UA | 2.901 | | | 2.318 | 0.120 | 0.011 | -0.317 |
| TCGA-FD-A62P | 3.801 | | | 2.092 | 0.157 | 0.222 | -0.311 |
| TCGA-XF-AAN4 | 7.511 | | | 1.078 | 0.063 | 0.083 | -0.309 |
| TCGA-YF-AA3M | 4.927 | | | 1.761 | 0.144 | 0.248 | -0.306 |
| TCGA-UY-A8OD | 3.443 | | | 2.088 | 0.129 | 0.040 | -0.306 |
| TCGA-ZF-AA4V | 4.077 | | | 1.988 | 0.136 | 0.401 | -0.304 |
| TCGA-BT-A20V | 12.168 | | | 0.104 | 1.108 | 0.611 | -0.304 |
| TCGA-FD-A5BT | 8.127 | | | 1.375 | 0.000 | 2.341 | -0.303 |
| TCGA-DK-A1AA | 3.626 | | | 2.000 | 0.182 | 0.023 | -0.302 |
| TCGA-XF-A9T2 | 3.646 | | | 2.233 | 0.777 | 0.866 | -0.297 |
| TCGA-DK-A1A7 | 1.649 | | | 2.423 | 0.093 | 0.000 | -0.295 |
| TCGA-FD-A3SM | 8.711 | | | 0.601 | 0.145 | 0.020 | -0.292 |
| TCGA-DK-A3IT | 2.638 | | | 2.243 | 0.166 | 0.462 | -0.290 |
| TCGA-CU-A72E | 1.485 | | | 2.475 | 0.534 | 0.000 | -0.289 |
| TCGA-FD-A5C0 | 1.580 | | | 2.333 | 0.152 | 0.034 | -0.282 |
| TCGA-BT-A0S7 | 5.654 | | | 1.294 | 0.013 | 0.159 | -0.281 |
| TCGA-G2-AA3C | 12.806 | | | 0.515 | 7.147 | 0.329 | -0.279 |
| TCGA-DK-A3X2 | 1.610 | | | 2.273 | 0.015 | 0.082 | -0.277 |
| TCGA-ZF-A9R1 | 1.744 | | | 2.209 | 0.112 | 0.000 | -0.275 |
| TCGA-K4-A4AB | 9.341 | | | 0.265 | 0.144 | 0.036 | -0.273 |
| TCGA-E7-A7DV | 9.547 | | | 0.535 | 0.088 | 1.491 | -0.273 |
| TCGA-BT-A20U | 28.211 | | | 0.077 | 32.281 | 0.035 | -0.270 |
| TCGA-BL-A13J | 7.343 | | | 0.674 | 0.089 | 0.000 | -0.265 |
| TCGA-FD-A5BZ | 2.607 | | | 1.850 | 0.064 | 0.057 | -0.260 |
| TCGA-G2-A2EO | 5.867 | | | 0.994 | 0.018 | 0.035 | -0.259 |
| TCGA-UY-A78M | 1.733 | | | 2.025 | 0.042 | 0.000 | -0.256 |
| TCGA-KQ-A41S | 4.084 | | | 1.346 | 0.112 | 0.011 | -0.247 |
| TCGA-DK-A3IL | 0.890 | | | 2.136 | 0.026 | 0.000 | -0.246 |
| TCGA-BT-A42F | 8.207 | | | 0.272 | 0.000 | 0.113 | -0.244 |
| TCGA-BT-A0YX | 6.626 | | | 0.624 | 0.579 | 0.023 | -0.232 |
| TCGA-4Z-AA84 | 1.124 | | | 2.093 | 0.078 | 0.709 | -0.230 |
| TCGA-FD-A5BU | 13.708 | | | 0.562 | 12.326 | 0.529 | -0.224 |
| TCGA-FD-A6TG | 3.388 | | | 1.276 | 0.000 | 0.019 | -0.223 |
| TCGA-DK-A2HX | 2.338 | | | 1.484 | 0.024 | 0.056 | -0.215 |
| TCGA-BT-A20R | 5.198 | | | 0.737 | 0.031 | 0.020 | -0.214 |
| TCGA-C4-A0F0 | 7.826 | | | 0.458 | 0.024 | 1.783 | -0.213 |
| TCGA-K4-A54R | 4.607 | | | 0.923 | 0.390 | 0.030 | -0.212 |
| TCGA-FD-A3SL | 3.740 | | | 1.080 | 0.025 | 0.089 | -0.210 |
| TCGA-ZF-AA56 | 19.373 | | | 0.770 | 26.190 | 0.023 | -0.199 |
| TCGA-DK-A3X1 | 4.754 | | | 0.706 | 0.061 | 0.000 | -0.199 |
| TCGA-BT-A20N | 2.056 | | | 1.346 | 0.022 | 0.000 | -0.195 |
| TCGA-XF-A9SV | 0.821 | | | 1.653 | 0.122 | 0.070 | -0.190 |
| TCGA-FT-A3EE | 2.338 | | | 1.247 | 0.136 | 0.130 | -0.187 |
| TCGA-BT-A3PH | 0.790 | | | 1.562 | 0.049 | 0.008 | -0.183 |
| TCGA-FJ-A871 | 1.238 | | | 1.352 | 0.077 | 0.826 | -0.153 |
| TCGA-XF-AAN8 | 3.817 | | | 0.359 | 0.000 | 0.184 | -0.135 |
| TCGA-DK-A1A3 | 2.739 | | | 0.597 | 0.077 | 0.038 | -0.133 |
| TCGA-FD-A6TF | 2.610 | | | 0.683 | 0.190 | 0.388 | -0.128 |
| TCGA-PQ-A6FN | 2.891 | | | 0.739 | 0.041 | 1.143 | -0.126 |
| TCGA-DK-A3IQ | 1.612 | | | 0.787 | 0.132 | 0.000 | -0.123 |
| TCGA-DK-AA6R | 1.389 | | | 0.844 | 0.148 | 0.049 | -0.121 |
| TCGA-ZF-AA4N | 11.057 | | | 0.267 | 24.124 | 0.155 | 0.046 |
| TCGA-G2-A3IB | 17.844 | | | 0.006 | 37.103 | 0.053 | 0.087 |
| TCGA-XF-AAN3 | 6.931 | | | 0.108 | 25.976 | 0.063 | 0.199 |
| TCGA-DK-A1AE | 13.007 | | | 2.036 | 56.283 | 0.172 | 0.298 |
| TCGA-GU-A42Q | 13.611 | | | 0.168 | 49.183 | 0.009 | 0.365 |
| TCGA-GU-AATQ | 8.476 | | | 0.667 | 45.489 | 0.023 | 0.394 |
| TCGA-BL-A13I | 9.381 | | | 3.059 | 63.939 | 1.009 | 0.424 |
| TCGA-K4-A5RI | 3.648 | | | 0.083 | 56.131 | 0.000 | 0.744 |
| TCGA-BT-A20J | 5.499 | | | 2.961 | 92.002 | 0.085 | 0.940 |
| TCGA-2F-A9KW | 2.440 | | | 4.202 | 0.031 | 76.072 | 1.343 |
| TCGA-XF-A9ST | 0.562 | | | 6.274 | 0.021 | 129.730 | 2.478 |
