# Supplementary material for: Identification of Immune-Related lncRNA Signature to Predict Prognosis and Immunotherapeutic Efficiency in Bladder Cancer
Source: Front Oncol. 2021 Jan 20;10:542140. doi: 10.3389/fonc.2020.542140 (PMC7855860; doi:10.3389/fonc.2020.542140)
Supplement: Supplementary file 4 [file Table_3.docx]

|  | risk | No benefits | Responder | TIDE |
| --- | --- | --- | --- | --- |
| TCGA-BL-A0C8 | low | FALSE | TRUE | -0.75 |
| TCGA-G2-AA3B | low | FALSE | TRUE | -1.29 |
| TCGA-ZF-A9R4 | low | FALSE | TRUE | -1.79 |
| TCGA-FJ-A3Z9 | low | FALSE | TRUE | -0.48 |
| TCGA-G2-A3IE | low | FALSE | TRUE | -0.85 |
| TCGA-GV-A6ZA | low | FALSE | TRUE | -0.41 |
| TCGA-ZF-A9R7 | low | FALSE | FALSE | 1.6 |
| TCGA-GC-A3I6 | low | FALSE | TRUE | -0.61 |
| TCGA-CF-A3MF | low | FALSE | TRUE | -1.59 |
| TCGA-E7-A3X6 | low | FALSE | FALSE | 0.65 |
| TCGA-FD-A43P | low | FALSE | FALSE | 0.62 |
| TCGA-2F-A9KO | low | FALSE | FALSE | 0.42 |
| TCGA-K4-A5RH | low | FALSE | FALSE | 0.8 |
| TCGA-UY-A9PB | low | FALSE | FALSE | 0.97 |
| TCGA-2F-A9KR | low | FALSE | TRUE | -0.34 |
| TCGA-E7-A6ME | low | FALSE | FALSE | 0.08 |
| TCGA-CF-A27C | low | FALSE | TRUE | -1.39 |
| TCGA-DK-A6B6 | low | FALSE | TRUE | -2.63 |
| TCGA-UY-A9PH | low | FALSE | FALSE | 1.33 |
| TCGA-ZF-A9RL | low | FALSE | TRUE | -0.69 |
| TCGA-CU-A3YL | low | FALSE | TRUE | -0.88 |
| TCGA-XF-AAML | low | FALSE | TRUE | -1.18 |
| TCGA-GC-A3WC | low | FALSE | FALSE | 0.43 |
| TCGA-ZF-A9R5 | low | FALSE | TRUE | -1.28 |
| TCGA-E7-A8O7 | low | FALSE | TRUE | -1.61 |
| TCGA-LC-A66R | low | FALSE | FALSE | 0.26 |
| TCGA-XF-A9SM | low | FALSE | FALSE | 0.98 |
| TCGA-E5-A4U1 | low | FALSE | TRUE | -0.37 |
| TCGA-XF-AAMX | low | FALSE | FALSE | 0.35 |
| TCGA-K4-A5RJ | low | FALSE | FALSE | 0.44 |
| TCGA-E7-A677 | low | FALSE | FALSE | 0.07 |
| TCGA-K4-A3WS | low | FALSE | FALSE | 1 |
| TCGA-XF-A8HD | low | FALSE | FALSE | 0.63 |
| TCGA-FJ-A3ZF | low | FALSE | TRUE | -0.27 |
| TCGA-FD-A3B6 | low | FALSE | FALSE | 0.46 |
| TCGA-GU-A766 | low | FALSE | FALSE | 1.4 |
| TCGA-E7-A6MF | low | FALSE | TRUE | -1.14 |
| TCGA-YF-AA3L | low | FALSE | TRUE | -0.35 |
| TCGA-G2-AA3F | low | FALSE | TRUE | -0.39 |
| TCGA-GU-A762 | low | FALSE | FALSE | 1.46 |
| TCGA-E7-A7XN | low | FALSE | FALSE | 1.05 |
| TCGA-PQ-A6FI | low | FALSE | TRUE | -0.43 |
| TCGA-ZF-AA4X | low | FALSE | TRUE | -1.23 |
| TCGA-GD-A76B | low | FALSE | FALSE | 0.76 |
| TCGA-GV-A3JX | low | FALSE | FALSE | 0.25 |
| TCGA-GV-A3JW | low | FALSE | TRUE | -0.1 |
| TCGA-G2-A2EK | low | FALSE | TRUE | -0.55 |
| TCGA-CF-A7I0 | low | FALSE | FALSE | 0.04 |
| TCGA-KQ-A41O | low | FALSE | TRUE | -0.34 |
| TCGA-CF-A9FH | low | FALSE | TRUE | -1.41 |
| TCGA-DK-A3WW | low | FALSE | FALSE | 0.24 |
| TCGA-XF-AAMZ | low | FALSE | TRUE | -0.09 |
| TCGA-G2-A2EJ | low | FALSE | TRUE | -0.69 |
| TCGA-E7-A678 | low | FALSE | TRUE | -1.52 |
| TCGA-4Z-AA7M | low | FALSE | FALSE | 0.6 |
| TCGA-ZF-A9RM | low | FALSE | TRUE | -0.81 |
| TCGA-ZF-AA58 | low | FALSE | FALSE | 0.95 |
| TCGA-FD-A3B4 | low | FALSE | FALSE | 0.68 |
| TCGA-DK-AA6W | low | FALSE | FALSE | 0.42 |
| TCGA-S5-AA26 | low | FALSE | FALSE | 0.07 |
| TCGA-CF-A47V | low | FALSE | TRUE | -1.07 |
| TCGA-BT-A3PJ | low | FALSE | TRUE | -0.15 |
| TCGA-GV-A3QI | low | FALSE | FALSE | 0.36 |
| TCGA-ZF-A9RF | low | FALSE | FALSE | 0.12 |
| TCGA-ZF-A9RC | low | TRUE | FALSE | 2.11 |
| TCGA-XF-AAMY | low | FALSE | FALSE | 0.56 |
| TCGA-FT-A61P | low | FALSE | FALSE | 0.92 |
| TCGA-XF-AAN1 | low | FALSE | TRUE | -0.31 |
| TCGA-DK-A3WY | low | FALSE | FALSE | 1.7 |
| TCGA-4Z-AA89 | low | FALSE | TRUE | -1.5 |
| TCGA-DK-A3IK | low | FALSE | FALSE | 0.21 |
| TCGA-GC-A3RB | low | FALSE | FALSE | 0.42 |
| TCGA-BT-A2LA | low | TRUE | FALSE | 1.27 |
| TCGA-XF-A8HC | low | FALSE | TRUE | -0.61 |
| TCGA-UY-A9PA | low | FALSE | FALSE | 0.73 |
| TCGA-UY-A78P | low | FALSE | FALSE | 0.87 |
| TCGA-LT-A8JT | low | FALSE | TRUE | -1.13 |
| TCGA-GU-AATP | low | FALSE | FALSE | 0.69 |
| TCGA-G2-AA3D | low | FALSE | TRUE | -0.97 |
| TCGA-4Z-AA7O | low | FALSE | FALSE | 0.02 |
| TCGA-CF-A47S | low | FALSE | TRUE | -1.56 |
| TCGA-UY-A9PD | low | FALSE | FALSE | 1.08 |
| TCGA-G2-A3VY | low | FALSE | TRUE | -0.56 |
| TCGA-DK-A3IV | low | FALSE | FALSE | 0.57 |
| TCGA-K4-A3WV | low | FALSE | FALSE | 0.67 |
| TCGA-GC-A3BM | low | FALSE | TRUE | -0.31 |
| TCGA-ZF-A9R2 | low | FALSE | TRUE | -0.45 |
| TCGA-GV-A3JV | low | FALSE | FALSE | 0.41 |
| TCGA-FD-A43X | low | FALSE | TRUE | -0.63 |
| TCGA-GV-A3QK | low | FALSE | FALSE | 0.38 |
| TCGA-XF-A9T5 | low | FALSE | FALSE | 0.92 |
| TCGA-4Z-AA7W | low | FALSE | FALSE | 1.47 |
| TCGA-DK-A3IS | low | FALSE | TRUE | -0.21 |
| TCGA-FD-A5BR | low | FALSE | FALSE | 0.24 |
| TCGA-LT-A5Z6 | low | FALSE | TRUE | -0.11 |
| TCGA-FD-A43S | low | FALSE | FALSE | 2.35 |
| TCGA-FD-A43Y | low | FALSE | FALSE | 0.68 |
| TCGA-FD-A6TE | low | FALSE | TRUE | -0.69 |
| TCGA-G2-A2EL | low | FALSE | FALSE | 0.19 |
| TCGA-ZF-A9R0 | low | FALSE | TRUE | -0.24 |
| TCGA-UY-A78K | low | FALSE | FALSE | 0.96 |
| TCGA-FJ-A3ZE | low | FALSE | TRUE | -0.41 |
| TCGA-FD-A3N6 | low | FALSE | TRUE | -0.22 |
| TCGA-XF-AAMG | low | FALSE | FALSE | 0.91 |
| TCGA-DK-A2I4 | low | FALSE | FALSE | 1.39 |
| TCGA-GU-A763 | low | FALSE | TRUE | -0.71 |
| TCGA-XF-AAN5 | low | FALSE | TRUE | -0.17 |
| TCGA-FD-A5C1 | low | FALSE | FALSE | 1.06 |
| TCGA-DK-A6AW | low | FALSE | TRUE | -0.66 |
| TCGA-E7-A4XJ | low | FALSE | TRUE | -0.19 |
| TCGA-DK-AA6X | low | FALSE | TRUE | -0.86 |
| TCGA-FD-A3N5 | low | FALSE | FALSE | 0.27 |
| TCGA-UY-A8OB | low | FALSE | FALSE | 0.05 |
| TCGA-G2-A2EC | low | FALSE | FALSE | 0.78 |
| TCGA-4Z-AA87 | low | FALSE | TRUE | -0.63 |
| TCGA-FD-A3B8 | low | FALSE | FALSE | 1.47 |
| TCGA-DK-AA76 | low | FALSE | TRUE | -0.34 |
| TCGA-4Z-AA86 | low | FALSE | FALSE | 0.73 |
| TCGA-DK-AA6L | low | FALSE | TRUE | -0.41 |
| TCGA-ZF-A9RD | low | FALSE | FALSE | 0.81 |
| TCGA-ZF-AA4R | low | FALSE | FALSE | 2.09 |
| TCGA-E7-A97Q | low | FALSE | TRUE | -0.02 |
| TCGA-E7-A85H | low | FALSE | TRUE | -0.59 |
| TCGA-XF-A9T4 | low | FALSE | FALSE | 1.02 |
| TCGA-FD-A3SR | low | FALSE | TRUE | -0.51 |
| TCGA-DK-AA6U | low | FALSE | FALSE | 0.07 |
| TCGA-GV-A3QH | low | FALSE | TRUE | -0.63 |
| TCGA-C4-A0F1 | low | FALSE | FALSE | 0.54 |
| TCGA-DK-A1A6 | low | FALSE | TRUE | -0.3 |
| TCGA-4Z-AA83 | low | FALSE | TRUE | -0.62 |
| TCGA-FD-A3SQ | low | FALSE | FALSE | 0.91 |
| TCGA-4Z-AA7N | low | FALSE | FALSE | 2.21 |
| TCGA-HQ-A5ND | low | FALSE | TRUE | -1.13 |
| TCGA-4Z-AA7Q | low | FALSE | FALSE | 0.99 |
| TCGA-DK-AA71 | low | FALSE | FALSE | 0.09 |
| TCGA-BT-A20W | low | FALSE | FALSE | 0.21 |
| TCGA-FD-A5BY | low | FALSE | FALSE | 1.05 |
| TCGA-E7-A97P | low | FALSE | FALSE | 0.74 |
| TCGA-E7-A3Y1 | low | FALSE | TRUE | -0.94 |
| TCGA-FD-A3B7 | low | FALSE | FALSE | 1.07 |
| TCGA-FD-A62N | low | FALSE | FALSE | 2.1 |
| TCGA-ZF-A9R3 | low | FALSE | TRUE | -1.06 |
| TCGA-YC-A8S6 | low | FALSE | FALSE | 0.25 |
| TCGA-BT-A2LB | low | FALSE | FALSE | 0.98 |
| TCGA-GU-A42P | low | FALSE | TRUE | -0.08 |
| TCGA-DK-AA74 | low | FALSE | FALSE | 1.67 |
| TCGA-CF-A5U8 | low | FALSE | TRUE | -0.87 |
| TCGA-HQ-A2OF | low | FALSE | TRUE | -0.48 |
| TCGA-GV-A3QF | low | FALSE | TRUE | -0.59 |
| TCGA-DK-A1AG | low | FALSE | TRUE | -0.73 |
| TCGA-FD-A6TB | low | FALSE | FALSE | 1.59 |
| TCGA-BT-A20T | low | FALSE | FALSE | 0.57 |
| TCGA-DK-A6B1 | low | FALSE | TRUE | -0.12 |
| TCGA-XF-A9T8 | low | FALSE | FALSE | 0.64 |
| TCGA-XF-A9SI | low | FALSE | FALSE | 1.64 |
| TCGA-FD-A62O | low | FALSE | FALSE | 0.49 |
| TCGA-XF-A9SK | low | FALSE | FALSE | 1.27 |
| TCGA-XF-A9T3 | low | FALSE | FALSE | 1 |
| TCGA-E7-A4IJ | low | FALSE | TRUE | -0.16 |
| TCGA-CU-A3KJ | low | FALSE | TRUE | -0.76 |
| TCGA-GD-A3OP | low | FALSE | FALSE | 0.47 |
| TCGA-DK-A3IU | low | FALSE | FALSE | 1.81 |
| TCGA-E7-A7PW | low | FALSE | TRUE | -1.01 |
| TCGA-K4-A6FZ | low | FALSE | FALSE | 0.49 |
| TCGA-FD-A3NA | low | FALSE | FALSE | 0.05 |
| TCGA-XF-AAN2 | low | FALSE | TRUE | -0.23 |
| TCGA-4Z-AA81 | low | FALSE | FALSE | 0.2 |
| TCGA-XF-AAMQ | low | FALSE | FALSE | 0.65 |
| TCGA-KQ-A41R | low | FALSE | TRUE | -0.93 |
| TCGA-H4-A2HO | low | FALSE | TRUE | -1.1 |
| TCGA-E7-A541 | low | FALSE | FALSE | 0.79 |
| TCGA-DK-A6B5 | low | FALSE | TRUE | -0.73 |
| TCGA-DK-AA77 | low | FALSE | TRUE | -0.38 |
| TCGA-BT-A20O | low | FALSE | FALSE | 1 |
| TCGA-UY-A78N | low | TRUE | FALSE | 1.39 |
| TCGA-FD-A5BV | low | TRUE | FALSE | 1.18 |
| TCGA-XF-A8HF | low | FALSE | FALSE | 0.72 |
| TCGA-SY-A9G0 | low | FALSE | FALSE | 1.63 |
| TCGA-FD-A5BS | low | FALSE | FALSE | 2.06 |
| TCGA-ZF-AA4U | low | FALSE | TRUE | -0.57 |
| TCGA-GV-A40G | low | FALSE | TRUE | -0.55 |
| TCGA-FD-A6TD | low | FALSE | FALSE | 0.81 |
| TCGA-E7-A519 | low | FALSE | TRUE | -1.19 |
| TCGA-K4-A3WU | low | FALSE | FALSE | 0.96 |
| TCGA-XF-A8HB | low | FALSE | TRUE | -0.39 |
| TCGA-ZF-AA4W | low | FALSE | FALSE | 0.14 |
| TCGA-CF-A1HR | low | FALSE | TRUE | -0.82 |
| TCGA-H4-A2HQ | low | FALSE | FALSE | 0.06 |
| TCGA-XF-A9SU | low | FALSE | FALSE | 1.1 |
| TCGA-DK-AA6M | low | FALSE | FALSE | 0.14 |
| TCGA-FD-A3SP | low | FALSE | FALSE | 0.95 |
| TCGA-BT-A20Q | low | FALSE | FALSE | 0.87 |
| TCGA-K4-A83P | low | FALSE | FALSE | 1.88 |
| TCGA-DK-A6B2 | low | FALSE | FALSE | 0.71 |
| TCGA-DK-AA6S | low | FALSE | FALSE | 0.97 |
| TCGA-CF-A47W | low | FALSE | TRUE | -0.72 |
| TCGA-ZF-AA51 | low | FALSE | FALSE | 0.67 |
| TCGA-XF-A8HI | low | FALSE | TRUE | -0.94 |
| TCGA-G2-A2EF | low | FALSE | TRUE | -0.01 |
| TCGA-E5-A4TZ | low | FALSE | TRUE | -0.84 |
| TCGA-FD-A3B3 | low | FALSE | FALSE | 0.81 |
| TCGA-GC-A3RC | low | FALSE | FALSE | 0.66 |
| TCGA-DK-A6B0 | low | FALSE | TRUE | -0.94 |
| TCGA-KQ-A41P | low | FALSE | FALSE | 0.33 |
| TCGA-ZF-AA53 | low | FALSE | FALSE | 0.55 |
| TCGA-CF-A47X | low | FALSE | TRUE | -1.28 |
| TCGA-K4-AAQO | low | FALSE | FALSE | 0.37 |
| TCGA-GD-A3OS | high | TRUE | FALSE | 2.02 |
| TCGA-DK-A3WX | high | FALSE | FALSE | 0.61 |
| TCGA-HQ-A5NE | high | FALSE | FALSE | 0.78 |
| TCGA-G2-A2ES | high | FALSE | TRUE | -0.53 |
| TCGA-GU-A767 | high | FALSE | FALSE | 0.87 |
| TCGA-CU-A0YO | high | FALSE | TRUE | -0.63 |
| TCGA-XF-A9SP | high | FALSE | FALSE | 1.84 |
| TCGA-C4-A0EZ | high | FALSE | FALSE | 0.58 |
| TCGA-4Z-AA7Y | high | FALSE | TRUE | -0.07 |
| TCGA-FD-A6TA | high | FALSE | FALSE | 0.12 |
| TCGA-5N-A9KM | high | FALSE | TRUE | -0.1 |
| TCGA-XF-A9SX | high | FALSE | FALSE | 1.44 |
| TCGA-DK-A1AB | high | FALSE | FALSE | 0.57 |
| TCGA-SY-A9G5 | high | FALSE | FALSE | 1.78 |
| TCGA-XF-AAMT | high | FALSE | FALSE | 1.79 |
| TCGA-XF-AAMR | high | TRUE | FALSE | 1.14 |
| TCGA-DK-AA6T | high | FALSE | FALSE | 0.81 |
| TCGA-FD-A6TK | high | FALSE | FALSE | 1.27 |
| TCGA-FJ-A3Z7 | high | FALSE | TRUE | -0.5 |
| TCGA-CF-A47Y | high | FALSE | TRUE | -1.16 |
| TCGA-BT-A20P | high | FALSE | FALSE | 0.29 |
| TCGA-5N-A9KI | high | FALSE | TRUE | -0.25 |
| TCGA-BL-A3JM | high | FALSE | TRUE | -0.43 |
| TCGA-XF-AAMH | high | TRUE | FALSE | 1.3 |
| TCGA-FD-A3SO | high | FALSE | FALSE | 0.08 |
| TCGA-XF-A9SJ | high | FALSE | FALSE | 0.92 |
| TCGA-CF-A8HX | high | FALSE | TRUE | -0.59 |
| TCGA-DK-A1A5 | high | FALSE | FALSE | 1.44 |
| TCGA-2F-A9KP | high | FALSE | FALSE | 0.19 |
| TCGA-ZF-AA5H | high | FALSE | FALSE | 0.5 |
| TCGA-UY-A78O | high | FALSE | TRUE | -0.4 |
| TCGA-XF-AAME | high | FALSE | FALSE | 1.41 |
| TCGA-CU-A5W6 | high | FALSE | TRUE | -0.5 |
| TCGA-XF-A9SY | high | FALSE | FALSE | 0.84 |
| TCGA-FD-A3B5 | high | FALSE | FALSE | 0.86 |
| TCGA-CF-A9FF | high | FALSE | TRUE | -0.81 |
| TCGA-GC-A3YS | high | FALSE | FALSE | 1.56 |
| TCGA-CF-A9FM | high | FALSE | TRUE | -1.72 |
| TCGA-DK-A3IN | high | FALSE | FALSE | 0.94 |
| TCGA-GU-A42R | high | FALSE | TRUE | -0.15 |
| TCGA-GV-A3JZ | high | FALSE | FALSE | 0.59 |
| TCGA-DK-AA6P | high | FALSE | TRUE | -0.48 |
| TCGA-ZF-AA4T | high | FALSE | TRUE | -0.32 |
| TCGA-DK-A1AD | high | FALSE | FALSE | 0.04 |
| TCGA-ZF-AA5P | high | FALSE | FALSE | 1.12 |
| TCGA-ZF-A9RN | high | FALSE | TRUE | -0.48 |
| TCGA-S5-A6DX | high | FALSE | FALSE | 1.46 |
| TCGA-GC-A3OO | high | FALSE | FALSE | 0.72 |
| TCGA-XF-A8HH | high | FALSE | FALSE | 0.34 |
| TCGA-4Z-AA82 | high | FALSE | FALSE | 1.07 |
| TCGA-XF-AAN0 | high | FALSE | FALSE | 0.62 |
| TCGA-CF-A3MI | high | FALSE | TRUE | -1.68 |
| TCGA-FD-A3SJ | high | FALSE | FALSE | 0.94 |
| TCGA-DK-A2I1 | high | FALSE | FALSE | 0.54 |
| TCGA-R3-A69X | high | FALSE | FALSE | 0.86 |
| TCGA-BT-A42E | high | FALSE | FALSE | 0.42 |
| TCGA-YC-A89H | high | FALSE | FALSE | 0.41 |
| TCGA-CF-A3MH | high | FALSE | TRUE | -0.55 |
| TCGA-GD-A6C6 | high | FALSE | TRUE | -0.41 |
| TCGA-GC-A3RD | high | FALSE | TRUE | -0.18 |
| TCGA-CF-A47T | high | FALSE | FALSE | 0.53 |
| TCGA-BT-A42C | high | FALSE | TRUE | -0.29 |
| TCGA-FD-A3SS | high | FALSE | FALSE | 0.31 |
| TCGA-KQ-A41Q | high | FALSE | TRUE | -0.05 |
| TCGA-HQ-A2OE | high | FALSE | TRUE | -1.01 |
| TCGA-ZF-AA54 | high | FALSE | FALSE | 0.41 |
| TCGA-E5-A2PC | high | FALSE | TRUE | -0.42 |
| TCGA-E7-A6MD | high | FALSE | TRUE | -0.27 |
| TCGA-4Z-AA7S | high | FALSE | TRUE | -0.13 |
| TCGA-K4-A4AC | high | FALSE | TRUE | -0.16 |
| TCGA-FD-A43N | high | FALSE | FALSE | 0.24 |
| TCGA-DK-A1AF | high | FALSE | FALSE | 1.34 |
| TCGA-XF-A9SL | high | FALSE | FALSE | 1.69 |
| TCGA-XF-AAMJ | high | FALSE | FALSE | 2.23 |
| TCGA-FD-A5BX | high | FALSE | FALSE | 0.65 |
| TCGA-MV-A51V | high | FALSE | TRUE | -0.28 |
| TCGA-2F-A9KQ | high | FALSE | FALSE | 0.05 |
| TCGA-UY-A9PE | high | FALSE | FALSE | 0.72 |
| TCGA-XF-A9T6 | high | FALSE | FALSE | 0.22 |
| TCGA-CF-A9FL | high | TRUE | FALSE | 1.02 |
| TCGA-CF-A1HS | high | FALSE | FALSE | 0.23 |
| TCGA-GU-A764 | high | FALSE | FALSE | 0.63 |
| TCGA-K4-A6MB | high | TRUE | FALSE | 1.1 |
| TCGA-XF-A8HG | high | FALSE | TRUE | -1.2 |
| TCGA-XF-AAN7 | high | FALSE | FALSE | 1.75 |
| TCGA-4Z-AA7R | high | FALSE | FALSE | 0.71 |
| TCGA-DK-AA75 | high | FALSE | FALSE | 0.01 |
| TCGA-KQ-A41N | high | FALSE | FALSE | 0.36 |
| TCGA-CU-A0YR | high | FALSE | TRUE | -1.2 |
| TCGA-2F-A9KT | high | FALSE | FALSE | 0.9 |
| TCGA-GD-A3OQ | high | FALSE | FALSE | 0.78 |
| TCGA-ZF-A9RE | high | FALSE | TRUE | -0.64 |
| TCGA-ZF-A9R9 | high | FALSE | FALSE | 0.78 |
| TCGA-CU-A3QU | high | FALSE | TRUE | -0.96 |
| TCGA-DK-AA6Q | high | FALSE | FALSE | 0.17 |
| TCGA-UY-A78L | high | FALSE | FALSE | 0.66 |
| TCGA-FD-A6TI | high | FALSE | TRUE | -0.13 |
| TCGA-XF-A9SH | high | FALSE | TRUE | -0.45 |
| TCGA-DK-A3IM | high | FALSE | FALSE | 0.67 |
| TCGA-BT-A20X | high | FALSE | TRUE | -0.32 |
| TCGA-CU-A0YN | high | FALSE | FALSE | 0.22 |
| TCGA-FD-A43U | high | FALSE | FALSE | 1.99 |
| TCGA-DK-A1AC | high | FALSE | TRUE | -0.73 |
| TCGA-GV-A40E | high | FALSE | FALSE | 0.77 |
| TCGA-GD-A2C5 | high | FALSE | FALSE | 0.45 |
| TCGA-FD-A6TC | high | TRUE | FALSE | 1.71 |
| TCGA-GU-AATO | high | FALSE | FALSE | 0.48 |
| TCGA-DK-A2I6 | high | FALSE | FALSE | 0.99 |
| TCGA-XF-AAMW | high | FALSE | FALSE | 0.83 |
| TCGA-BL-A5ZZ | high | FALSE | FALSE | 1.63 |
| TCGA-ZF-AA52 | high | FALSE | FALSE | 0.81 |
| TCGA-DK-A2I2 | high | FALSE | FALSE | 0.78 |
| TCGA-BT-A3PK | high | FALSE | FALSE | 0.62 |
| TCGA-FD-A3SN | high | FALSE | FALSE | 1.02 |
| TCGA-ZF-AA5N | high | FALSE | TRUE | -0.1 |
| TCGA-XF-A9SW | high | FALSE | FALSE | 1.39 |
| TCGA-CF-A3MG | high | FALSE | TRUE | -0.74 |
| TCGA-XF-A9SZ | high | FALSE | FALSE | 0.72 |
| TCGA-FD-A6TH | high | FALSE | FALSE | 0.24 |
| TCGA-FD-A62S | high | FALSE | FALSE | 1.45 |
| TCGA-XF-A9T0 | high | FALSE | TRUE | -0.41 |
| TCGA-BT-A2LD | high | FALSE | FALSE | 0.35 |
| TCGA-DK-A6AV | high | FALSE | TRUE | -0.35 |
| TCGA-C4-A0F7 | high | FALSE | FALSE | 0.63 |
| TCGA-UY-A9PF | high | FALSE | TRUE | -0.5 |
| TCGA-C4-A0F6 | high | FALSE | FALSE | 0.7 |
| TCGA-CF-A8HY | high | FALSE | TRUE | -0.02 |
| TCGA-XF-A8HE | high | FALSE | FALSE | 0.28 |
| TCGA-CF-A5UA | high | FALSE | FALSE | 0.2 |
| TCGA-FD-A62P | high | FALSE | FALSE | 0.77 |
| TCGA-XF-AAN4 | high | FALSE | FALSE | 0.92 |
| TCGA-YF-AA3M | high | FALSE | FALSE | 0.57 |
| TCGA-UY-A8OD | high | FALSE | FALSE | 0.62 |
| TCGA-ZF-AA4V | high | FALSE | TRUE | -0.25 |
| TCGA-BT-A20V | high | FALSE | TRUE | -0.38 |
| TCGA-FD-A5BT | high | FALSE | FALSE | 1.8 |
| TCGA-DK-A1AA | high | FALSE | TRUE | -0.5 |
| TCGA-XF-A9T2 | high | TRUE | FALSE | 1.01 |
| TCGA-DK-A1A7 | high | FALSE | TRUE | -0.65 |
| TCGA-FD-A3SM | high | FALSE | FALSE | 1.08 |
| TCGA-DK-A3IT | high | FALSE | FALSE | 0.28 |
| TCGA-CU-A72E | high | FALSE | FALSE | 0.55 |
| TCGA-FD-A5C0 | high | FALSE | FALSE | 0.44 |
| TCGA-BT-A0S7 | high | FALSE | FALSE | 0.92 |
| TCGA-G2-AA3C | high | FALSE | FALSE | 0.07 |
| TCGA-DK-A3X2 | high | FALSE | FALSE | 0.07 |
| TCGA-ZF-A9R1 | high | FALSE | FALSE | 0.57 |
| TCGA-K4-A4AB | high | FALSE | FALSE | 0.9 |
| TCGA-E7-A7DV | high | FALSE | FALSE | 0.37 |
| TCGA-BT-A20U | high | FALSE | FALSE | 0.62 |
| TCGA-BL-A13J | high | FALSE | FALSE | 1.17 |
| TCGA-FD-A5BZ | high | FALSE | FALSE | 2.6 |
| TCGA-G2-A2EO | high | FALSE | FALSE | 0.21 |
| TCGA-UY-A78M | high | TRUE | FALSE | 1.4 |
| TCGA-KQ-A41S | high | FALSE | FALSE | 1.11 |
| TCGA-DK-A3IL | high | FALSE | FALSE | 0.59 |
| TCGA-BT-A42F | high | FALSE | FALSE | 0.73 |
| TCGA-BT-A0YX | high | FALSE | FALSE | 0.06 |
| TCGA-4Z-AA84 | high | FALSE | FALSE | 0.4 |
| TCGA-FD-A5BU | high | FALSE | FALSE | 0.49 |
| TCGA-FD-A6TG | high | FALSE | FALSE | 0.62 |
| TCGA-DK-A2HX | high | TRUE | FALSE | 1.27 |
| TCGA-BT-A20R | high | FALSE | FALSE | 1.31 |
| TCGA-C4-A0F0 | high | FALSE | TRUE | -0.89 |
| TCGA-K4-A54R | high | FALSE | FALSE | 0.7 |
| TCGA-FD-A3SL | high | FALSE | FALSE | 0.85 |
| TCGA-ZF-AA56 | high | FALSE | TRUE | -0.11 |
| TCGA-DK-A3X1 | high | FALSE | FALSE | 0.38 |
| TCGA-BT-A20N | high | FALSE | FALSE | 1.11 |
| TCGA-XF-A9SV | high | TRUE | FALSE | 1.09 |
| TCGA-FT-A3EE | high | FALSE | FALSE | 0.98 |
| TCGA-BT-A3PH | high | FALSE | TRUE | -0.23 |
| TCGA-FJ-A871 | high | TRUE | FALSE | 2.57 |
| TCGA-XF-AAN8 | high | FALSE | FALSE | 1.13 |
| TCGA-DK-A1A3 | high | FALSE | TRUE | -0.01 |
| TCGA-FD-A6TF | high | FALSE | FALSE | 1.77 |
| TCGA-PQ-A6FN | high | FALSE | FALSE | 0.79 |
| TCGA-DK-A3IQ | high | TRUE | FALSE | 2.72 |
| TCGA-DK-AA6R | high | FALSE | FALSE | 0.59 |
| TCGA-ZF-AA4N | high | FALSE | TRUE | -0.31 |
| TCGA-G2-A3IB | high | FALSE | TRUE | -0.42 |
| TCGA-XF-AAN3 | high | FALSE | FALSE | 0.04 |
| TCGA-DK-A1AE | high | FALSE | FALSE | 0.54 |
| TCGA-GU-A42Q | high | TRUE | FALSE | 1.04 |
| TCGA-GU-AATQ | high | FALSE | FALSE | 0.43 |
| TCGA-BL-A13I | high | FALSE | FALSE | 0.1 |
| TCGA-K4-A5RI | high | FALSE | FALSE | 1.11 |
| TCGA-BT-A20J | high | FALSE | FALSE | 0.39 |
| TCGA-2F-A9KW | high | FALSE | TRUE | -0.18 |
| TCGA-XF-A9ST | high | FALSE | FALSE | 0.7 |
