# Supplementary material for: Identification of Immune-Related lncRNA Signature to Predict Prognosis and Immunotherapeutic Efficiency in Bladder Cancer
Source: Front Oncol. 2021 Jan 20;10:542140. doi: 10.3389/fonc.2020.542140 (PMC7855860; doi:10.3389/fonc.2020.542140)
Supplement: Supplementary file 5 [file Table_4.docx]

|  | | | | | | | | |
| --- | --- | --- | --- | --- | --- | --- | --- | --- |
| ID | futime | fustat | Age | Gender | T | N | Grade | riskScore |
| 9 | 720 | 0 | 81 | 1 | 1 | 0 | 1 | -7.68562 |
| 47 | 600 | 1 | 76 | 0 | 2 | 0 | 1 | -0.07222 |
| 42 | 270 | 1 | 78 | 0 | 2 | 0 | 2 | -0.26088 |
| 6 | 960 | 0 | 54 | 1 | 2 | 0 | 1 | -10.8729 |
| 20 | 1558 | 0 | 55 | 1 | 2 | 0 | 1 | -3.34553 |
| 1 | 780 | 0 | 58 | 1 | 2 | 0 | 1 | -51.2133 |
| 27 | 1548 | 0 | 70 | 1 | 2 | 0 | 1 | -2.11541 |
| 19 | 807 | 0 | 63 | 0 | 3 | 0 | 1 | -3.64893 |
| 31 | 986 | 1 | 63 | 0 | 3 | 0 | 1 | -1.08465 |
| 4 | 720 | 0 | 64 | 0 | 3 | 0 | 1 | -13.6225 |
| 51 | 459 | 0 | 79 | 0 | 3 | 0 | 1 | -0.00801 |
| 37 | 429 | 0 | 49 | 1 | 3 | 0 | 1 | -0.43801 |
| 8 | 985 | 0 | 52 | 1 | 3 | 0 | 1 | -8.47909 |
| 14 | 1154 | 0 | 67 | 1 | 3 | 0 | 1 | -4.91393 |
| 30 | 450 | 0 | 75 | 1 | 3 | 0 | 1 | -1.51275 |
| 36 | 360 | 1 | 76 | 1 | 3 | 0 | 1 | -0.48417 |
| 15 | 780 | 1 | 60 | 0 | 4 | 0 | 2 | -4.34236 |
| 50 | 645 | 0 | 54 | 1 | 4 | 0 | 1 | -0.01845 |
| 28 | 1338 | 0 | 67 | 1 | 4 | 0 | 2 | -1.5975 |
| 24 | 748 | 0 | 49 | 0 | 1 | 1 | 1 | -2.31061 |
| 44 | 687 | 0 | 56 | 1 | 1 | 1 | 1 | -0.18178 |
| 25 | 1030 | 0 | 38 | 0 | 2 | 1 | 1 | -2.29453 |
| 12 | 580 | 0 | 76 | 0 | 2 | 1 | 1 | -6.70969 |
| 5 | 1366 | 0 | 54 | 1 | 2 | 1 | 1 | -10.9544 |
| 17 | 1238 | 0 | 66 | 1 | 2 | 1 | 1 | -3.85572 |
| 39 | 540 | 1 | 67 | 1 | 2 | 1 | 1 | -0.33443 |
| 10 | 946 | 1 | 74 | 1 | 2 | 1 | 1 | -7.61158 |
| 46 | 630 | 0 | 48 | 1 | 2 | 1 | 2 | -0.10482 |
| 34 | 996 | 1 | 53 | 1 | 2 | 1 | 2 | -0.72442 |
| 18 | 1045 | 0 | 49 | 0 | 3 | 1 | 1 | -3.84824 |
| 26 | 900 | 1 | 65 | 1 | 3 | 1 | 1 | -2.22886 |
| 23 | 359 | 1 | 68 | 1 | 3 | 1 | 1 | -2.70735 |
| 38 | 780 | 0 | 34 | 1 | 3 | 1 | 2 | -0.40611 |
| 7 | 1023 | 1 | 69 | 0 | 4 | 1 | 2 | -10.311 |
| 29 | 810 | 0 | 57 | 1 | 1 | 2 | 1 | -1.54234 |
| 52 | 840 | 1 | 83 | 1 | 2 | 2 | 1 | 0.032779 |
| 21 | 542 | 0 | 50 | 1 | 2 | 2 | 2 | -2.85852 |
| 40 | 559 | 1 | 59 | 0 | 3 | 2 | 1 | -0.28399 |
| 2 | 956 | 0 | 79 | 0 | 3 | 2 | 1 | -25.2667 |
| 43 | 360 | 1 | 49 | 1 | 3 | 2 | 2 | -0.20791 |
| 3 | 1568 | 0 | 63 | 1 | 3 | 2 | 2 | -20.5704 |
| 45 | 166 | 1 | 68 | 1 | 3 | 2 | 2 | -0.17676 |
| 41 | 330 | 1 | 68 | 1 | 3 | 2 | 2 | -0.26721 |
| 16 | 520 | 1 | 70 | 1 | 3 | 2 | 2 | -4.17199 |
| 11 | 420 | 0 | 73 | 1 | 3 | 2 | 2 | -7.47592 |
| 53 | 450 | 1 | 60 | 1 | 4 | 2 | 2 | 0.119804 |
| 35 | 365 | 1 | 65 | 1 | 4 | 2 | 2 | -0.51872 |
| 13 | 930 | 1 | 79 | 1 | 4 | 2 | 2 | -6.35941 |
| 32 | 320 | 1 | 69 | 0 | 3 | 3 | 2 | -0.81614 |
| 33 | 660 | 0 | 80 | 0 | 3 | 3 | 2 | -0.79178 |
| 54 | 210 | 1 | 66 | 1 | 3 | 3 | 1 | 0.616081 |
| 48 | 640 | 0 | 36 | 1 | 3 | 3 | 2 | -0.05153 |
| 49 | 540 | 1 | 58 | 0 | 4 | 3 | 2 | -0.03649 |
| 22 | 685 | 1 | 61 | 1 | 4 | 3 | 1 | -2.71047 |
